# Comprehensive analysis of the BC200 ribonucleoprotein reveals a reciprocal regulatory function with CSDE1/UNR

**DOI:** 10.1093/nar/gky860

**Published:** 2018-09-22

**Authors:** Evan P Booy, Ewan KS McRae, Peyman Ezzati, Taegi Choi, Daniel Gussakovsky, Sean A McKenna

**Affiliations:** 1 Department of Chemistry, University of Manitoba, Winnipeg, Manitoba, Canada; 2 Manitoba Centre for Proteomics and Systems Biology, Section of Biomedical Proteomics, Department of Internal Medicine, Rady Faculty of Health Sciences, University of Manitoba and Health Sciences Centre, Winnipeg, Manitoba, Canada; 3 Department of Biochemistry & Medical Genetics, University of Manitoba, Winnipeg, Manitoba, Canada

## Abstract

BC200 is a long non-coding RNA primarily expressed in brain but aberrantly expressed in various cancers. To gain a further understanding of the function of BC200, we performed proteomic analyses of the BC200 ribonucleoprotein (RNP) by transfection of 3′ DIG-labelled BC200. Protein binding partners of the functionally related murine RNA BC1 as well as a scrambled BC200 RNA were also assessed in both human and mouse cell lines. Stringent validation of proteins identified by mass spectrometry confirmed 14 of 84 protein binding partners and excluded eight proteins that did not appreciably bind BC200 in reverse experiments. Gene ontology analyses revealed general roles in RNA metabolic processes, RNA processing and splicing. Protein/RNA interaction sites were mapped with a series of RNA truncations revealing three distinct modes of interaction involving either the 5′ Alu-domain, 3′ A-rich or 3′ C-rich regions. Due to their high enrichment values in reverse experiments, CSDE1 and STRAP were further analyzed demonstrating a direct interaction between CSDE1 and BC200 and indirect binding of STRAP to BC200 via heterodimerization with CSDE1. Knock-down studies identified a reciprocal regulatory relationship between CSDE1 and BC200 and immunofluorescence analysis of BC200 knock-down cells demonstrated a dramatic reorganization of CSDE1 into distinct nuclear foci.

## INTRODUCTION

BC200 (brain cytoplasmic RNA 1, BCYRN1) is a primate-specific long non-coding RNA that is normally expressed at high levels in the brain but is aberrantly expressed in a wide variety of tumour types (1–7). BC200 demonstrates a similar expression pattern to the murine BC1 RNA, exhibiting elevated neuronal levels and dendritic localization (8). Functionally, both RNAs have been implicated as inhibitors of mRNA translation in both *in-vivo* and *in-vitro* translation assays; however, limited data exist concerning the molecular mechanisms and specific mRNA targets regulated by BC200 (9–12).

The BC200 RNA can be divided into three distinct segments, the first consisting of 120 nucleotides that are homologous to the left monomer of Alu-J repetitive elements (Alu domain), the second a central 40 nucleotide adenosine rich stretch and the third, a unique 3′ region of 40 nucleotides that also possesses a continuous run of 12 cytosines (8,13,14). The BC1 RNA on the other hand exhibits little sequence similarity to BC200 with the exception of a ∼50 nucleotide adenosine rich stretch (8). Despite a lack of sequence homology, all studies to date have confirmed similar expression patterns and functional outcomes of these RNAs, indicating they likely fulfill analogous roles in distinct species.

BC200 has been clearly demonstrated to be critical for tumour cell viability as well as cell migration and invasion (1,5,7,15,16). In a neuronal context, two studies suggest altered expression patterns in neurodegenerative disease and aging (17,18). Despite well defined knock-down phenotypes in tumour cell culture assays as well as murine xenograft models, a thorough understanding of the cellular mechanism of BC200 remains elusive. In tumour cells, BC200 is primarily localized to the cytoplasm where it has been shown to bind a number of proteins (SRP9/SRP14, PABPC1, eIF4A, FMR1, SYNCRIP, hnRNPA2B1, PCBP1/2 and DHX36); however, a complete analysis of the BC200 ribonucleoprotein (RNP) has yet to be performed (7,9,10,19–24). Furthermore, only a small number of mRNAs (BCL-X, S100A11, MMP9/13) have been identified as BC200 targets (5,7,16). As such, comprehensive analysis of the BC200 interacting proteins and mRNAs would shed significant light onto the mechanism by which BC200 confers proliferative and invasive potential on cancer cells.

CSDE1 (cold shock domain-containing E1), also known as UNR, is a cytoplasmic RNA binding protein with high affinity for purine rich single stranded nucleic acids (25,26). CSDE1 is implicated in many facets of post-transcriptional gene regulation, having been demonstrated to both positively and negatively modulate mRNA stability and both stimulate and repress mRNA translation in a context dependent manner (27,28). Furthermore, CSDE1 regulates cap-independent translation during mitosis as well as under conditions of viral infection (29–32). In terms of cellular function, CSDE1 plays key roles in development, differentiation, apoptosis as well as cell migration (28,33–35). Underlining the importance of CSDE1 in human disease, a comprehensive recent study identified a key role for CSDE1 in melanoma as a regulator of a subset of genes governing cell invasion and metastasis (27).

While CSDE1 is primarily cytoplasmic, a recent report by Saltel *et al.* identifies a novel role for CSDE1 in mRNA translation control in the nucleoplasmic reticulum (NR) of polyploid cells referred to as the UNR-rich NR (36). CSDE1 knock-out was embryonic lethal in mice due to defects in placental development. While under normal conditions these UNR-rich NRs were only detectable in polyploid cells of the placenta and liver, they could also be induced in drug treated cancer cell lines. These CSDE1-rich nucleo-cytoplasmic interfaces contain both actively translated and repressed mRNAs (36).

CSDE1 interacts with the WD40 domain-containing protein STRAP (serine/threonine kinase receptor associated protein), also known as UNRIP (UNR interacting protein) (29). STRAP is a scaffolding protein that does not have inherent RNA-binding capability but has been shown to interact with RNA indirectly via protein binding partners (37). STRAP plays a role in assembly of the survival of motor neuron complex (SMN), a complex involved in the biogenesis of a variety of cellular ribonucleoproteins (37–39). STRAP interacts with the SMN complex via direct binding to Gemin7, an interaction that is mutually exclusive with CSDE1 binding. STRAP knockdown by siRNA results in an elevated number of nuclear coiled bodies/nuclear gems, indicating a role in the cellular distribution of the SMN complex (37,39). STRAP is frequently overexpressed in various cancers and has been shown to regulate translation by interacting with eIF4A (40–43). Despite their well-established interaction, the functional relationship between CSDE1 and STRAP remains poorly understood.

In this current work, we set out to gain insight into the function of the long non-coding RNA BC200 by identifying the protein binding partners within the BC200 RNP. To do this, we captured biologically relevant complexes assembled in cell with transfected DIG-labelled BC200. These protein binding partners were then subsequently validated by reverse immunoprecipitation experiments and by assessing binding to a scrambled control RNA. To evaluate the degree of functional overlap between primate BC200 and rodent BC1, we also performed similar experiments with BC1 in both human and murine cell lines. Stringent validation yielded a confirmed list of 14 BC200 binding proteins of which eight proteins were novel interaction partners. Control experiments ruled out eight of the 22 identified proteins as non-specific interactions as they did not bind endogenous BC200 in reverse experiments.

Binding experiments performed with 24 of the identified proteins and various truncations of the BC200 RNA defined three distinct modes of interaction with BC200 that were dependent upon either the 5′ Alu domain or 3′ A-rich or C-rich regions. Due to their high enrichment values, CSDE1 and STRAP were further investigated revealing coordinated binding that was CSDE1-dependent. The expression level of both BC200 and CSDE1 was interdependent, with a reduced half-life of BC200 observed in the absence of CSDE1 and markedly reduced CSDE1 expression upon BC200 knock-down. Finally, BC200 knock-down resulted in reorganization of CSDE1 into concentrated nuclear foci that were frequently associated with coiled bodies. These data provide a comprehensive list of BC200 and BC1 binding proteins in several cell lines that will serve as a basis for refining the molecular mechanisms of these long non-coding RNAs. Functional relationships observed between BC200 and CSDE1 serve to substantiate the proteomic screen and lay the groundwork for more detailed investigation into the mechanism by which BC200 and CSDE1 coordinate to regulate gene expression to support oncogenesis.

## MATERIALS AND METHODS

### Cell culture and reagents

The HEK293T cell line was a gift from Dr. Thomas Klonisch; the MCF-7 and MDA-MB-231 cell lines were a gift from Dr Spencer Gibson and the MEF cell line was a gift from Dr Peter Pelka. Cell culture conditions were as previously published (14). DNA primers and RNA oligonucleotides were purchased from Integrated DNA Technologies (Coralville, IA, USA). LNA GapmeRs were purchased from Exiqon (Woburn, MA, USA). siRNAs and non-targeting controls were purchased from Thermo Fisher Scientific (Ottawa, Canada). The plasmid for overexpression of BC200 under control of the U6 snRNA promoter was synthesized by Genscript Inc. (Piscataway, NJ, USA). The plasmid for BC200 expression from the endogenous promoter was generating by amplifying and cloning a 3788 nt fragment of the genomic sequence of the BC200 gene (–2314 to +1474) into the pJET1.2 vector. All standard laboratory chemicals and reagents were purchased from Thermo Fisher Scientific.

### *In-vitro* transcription, RNA purification and 3′ labelling

*In-vitro* transcription and purification of BC200, BC1, BCSCR, 7SL and indicated BC200 truncations was performed as previously described (44). Plasmids containing RNA sequence with a 5′ T7 promoter and 3′ linearization site were synthesized by Genscript Inc. For RNA pull-down assays, RNAs were ligated to a 5′ phosphorylated RNA linker containing a 3′ DIG label. The linker sequence is as follows: ACGUA-DIG. RNA ligation was performed with T4 RNA Ligase (New England Biolabs, Ipswich, MA) according to the manufacturer's protocol. Labeled RNA was subsequently purified using the GeneJet RNA Cleanup and Concentration Micro Kit (Thermo Fisher Scientific). Labeling efficiency was monitored by performing electrophoretic mobility shift assays (EMSAs) with an anti-DIG antibody (200–002-156, Jackson Immunologicals, West Grove, PA, USA).

### BC200 transfection and anti-DIG magnetic beads preparation

DIG-labelled BC200, BC1 and scrambled BC200 (BCSCR) were transfected into mammalian cells using Lipofectamine RNAiMax according to the manufacturer's protocol (Thermo-Fisher Scientific). 150 pmol of RNA were used per 150 mm tissue culture dish with 112.5 μl of Lipofectamine RNAiMax reagent. Cells were ∼75% confluent at time of transfection.

Crosslinking of anti-Dig IgG (Jackson Immunologicals) to protein A/G magnetic beads (Thermo-Fisher Scientific) was performed as follows: 700 μl protein A/G beads were equilibrated in PBS and incubated with 400 μg anti-Dig IgG for 1 h at 4°C with end over end mixing. Beads were subsequently equilibrated in 0.2 M triethanolamine (pH 8.2) and then resuspended in 0.2 M triethanolamine (pH 8.2) containing 25 mM dimethyl pimelimidate (DMP). Fresh DMP solution was replaced every 15 min for 45 min with end-over-end mixing followed by two washes with 0.1 M ethanolamine pH 8.2 and incubation in 0.1 M ethanolamine for 30 min at room temperature with end-over-end mixing. Beads were washed twice in PBS, followed by two washes in 0.1 M glycine pH 2.5 and finally washed threefold and stored in PBS containing 0.1% Tween and 0.02% sodium azide.

### RNP capture and on-bead trypsin digestion

Twenty four hours post-transfection, cells were scraped into 10 ml cold PBS per 150 mm dish and centrifuged at 500×g for 5 min. The cell pellet was resuspended in cold PBS and recentrifuged. Cytoplasmic extracts were prepared by resuspending cell pellets in 25 mM Hepes, pH 7.9, 5 mM KCl, 0.5 mM MgCl_2_, 0.5% (v/v) NP-40 supplemented with protease and RNase inhibitors (Halt protease and phosphatase inhibitor cocktail and Ribolock RNase inhibitor, Thermo-Fisher Scientific) followed by a 10-minute incubation at 4°C with end over end mixing. Following incubation, the buffer composition was adjusted to 25 mM HEPES pH 7.9, 5 mM KCl, 0.5 mM MgCl_2_, 0.25% (v/v) NP-40, 100 mM NaCl (IP Buffer) and insoluble material removed by centrifugation at 5000 rpm for 5 min in a bench top microcentrifuge at 4°C. Protein concentration was assessed by the standard Bradford assay and all lysate concentrations were normalized to a protein concentration of 5 mg/ml. 500 μl of lysate was used per immunoprecipitation. To capture BC200 RNP complexes, 50 μl of pre-equilibrated Protein A/G magnetic beads cross-linked to anti-Dig IgG were added to the lysate followed by end-over-end mixing at 4°C for 2 h. Following incubation, beads were washed 4-fold in IP Buffer. Bead-bound RNA–protein complexes were washed three times with 1 ml 50 mM ammonium bicarbonate and resuspended into Siliconized vials (BioPlas, San Rafael, CA, USA) in 50 μl of 50 mM ammonium bicarbonate and the proteins were reduced by 10 mM DTT at 50°C for 30 min. The proteins were alkylated with 30 mM iodoacetamide for 30 min in the dark at room temperature. Unreacted iodoacetamide was quenched by addition of 20 mM DTT. Finally, the protein complexes were digested by 500 ng of sequencing grade Trypsin (Promega) overnight at 37°C using a tube roller with gentle horizontal mixing. The reaction was stopped using 1% trifluoroacetic acid (TFA) by adding 50 μl of 3% TFA to the peptide–beads mixture. The beads were vortexed for 10 min and peptides were extracted. In order to maximize peptide-yield from each sample, we performed two sequential extractions using 200 μl of 0.1% TFA in acetonitrile, and 20 mM ammonium formate (pH 11) in acetonitrile. Peptides from each bead wash were pooled and dried using speed-vac. The dried peptides were dissolved in 100 μl of 0.5% TFA and desalted with 1 ml C18-SD extraction disc cartridge (3M, USA). 2 μg of desalted peptide as determined by NanoDrop 2000 (Thermo-Fisher) was used for LC–MS/MS analysis.

### Nano-RP–LC–MS/MS

Samples were analyzed by nano-RP–LC–MS/MS using a splitless Ultra 2D Plus (Eksigent, Dublin, CA, USA) system coupled to a high-speed Triple TOF™ 5600+ mass spectrometer (SCIEX, Concord, Canada). Peptides were injected via a PepMap100 trap column [0.3 × 5 mm, 5 μm, 100 Å, (Thermo Scientfic, CA, USA) and a 100 μm × 200 mm analytical column packed with 3 μm Luna C18 (Phenomenex Inc., CA, USA) was used prior to MS/MS analysis. Both eluents A (LC–MS water), and B (LC–MS acetonitrile) contained 0.1% formic acid as an ion-pairing modifier. The tryptic digest was analyzed using 90 min LC–MS run time. Eluent B had a gradient from 0% to 35% over 78 min, 35–85% in 1 min and was kept at 85% for 5 min at a flow rate of 500 nl/min. Key parameter settings for the TripleTOF 5600+ mass spectrometer were as follows: ion spray voltage floating (ISVF) 3000 V, curtain gas (CUR) 25, interface heater temperature (IHT) 150, ion source gas 1 (GS1) 25, declustering potential (DP) 80 V. All data were acquired using information-dependent acquisition (IDA) mode with Analyst TF1.6 software (SCIEX,USA). 0.25 s MS survey scan in the mass range of 380–1250 (*m/z*) were followed by 15 MS/MS scans of 150 ms in the mass range of 100–1600 (total cycle time: 2.5 s). Switching criteria were set to ions greater than mass to charge ratio (*m/z*) 380 and smaller than *m/z* 1250 with a charge state of +2 to +5 and an abundance threshold of >150 counts. Former target ions were excluded for 7 s. A sweeping collision energy setting of 37 ± 15 eV was applied to all precursor ions for collision-induced dissociation.

### Database search and protein identification

WIFF files containing MS and MS/MS data were analyzed using Protein Pilot 4.5 software using Paragon algorithm (SCIEX). Protein identification parameter of carbamidomethylation of cysteine was selected. Samples were searched against the curated UniProt's human proteome or mouse proteome release (2017_02) containing protein entries for unique canonical sequence and splice isoforms. Proteins with minimum of two unique peptides identified at >95% confidence (unused score > 1.3) were used for subsequent comparative quantitative analysis.

### SDS/PAGE, western blotting and antibodies

SDS/PAGE and western blotting were performed as previously described (45). Subcellular fractionation was performed with the Thermo-Scientific Subcellular Fraction Kit for Cultured cells according to the manufacturer's protocol (Thermo-Fisher Scientific). The following antibodies were used: Rabbit anti-DHX9 (ab26271, Abcam), Rabbit anti-DHX9 (ab70777, Abcam), Rabbit anti-PABP (ab21060, Abcam) Mouse anti-PABP (ab6125, Abcam), Rabbit anti-CSDE1 (ab201688, Abcam), Rabbit anti-CSDE1 (HPA052221, Sigma-Aldrich), Rabbit anti-CSDE1 (ab200663, Abcam), Rabbit anti-TRIM25 (ab86365, Abcam), Rabbit anti-TRIM25 (ab167154, Abcam), Rabbit anti-FAM120A (ab156695, Abcam), Rabbit anti-SYNCRIP (8588S, CST), Rabbit anti-SYNCRIP (14024-1-AP, Proteintech), Mouse anti-SYNCRIP (ab10687, Abcam), Rabbit anti-PABCP4 (14960-1-AP, Proteintech), Rabbit anti-PABC4 (ab220832, Abcam), Rabbit anti-ZC3HAV1 (16820-1-AP), Rabbit anti-DDX58 (3743S, CST), Mouse anti-DHX36 (Clone 12F33, made in-house), Rabbit anti-Nucleolin (14574, Cell Signalling), Rabbit anti-PCBP2 (ab184962, Abcam), Mouse anti-HRNPNK (ab39975, Abcam), Rabbit anti-HNRNPUL1 (ab68480, Abcam), Rabbit anti-DDX5 (ab126730, Abcam), Rabbit anti-SRP9 (11195-1-AP, Proteintech), Rabbit anti-SRP14 (11528-1-AP, Proteintech), Rabbit anti-PABPN1 (ab75855, Abcam), Mouse anti-Tubulin (T6074, Sigma-Alrich), Rabbit anti-DDX6 (ab70455, Abcam), Rabbit anti-STAU1 (14225-1-AP, Proteintech), Rabbit anti-STAU2 (15998-1-AP, Proteintech), Rabbit anti-STRAP (18277-1-AP, Proteintech), Rabbit anti-PARP12 (A305–130A, Bethyl Laboratories Inc., Montgomery, TX, USA), Rabbit anti-FMR1 (F4055, Sigma-Aldrich) Rabbit anti-c-Myc (13987T, Cell Signaling Technologies (CST)), Rabbit anti-LRP5 (5731S, CST), Goat anti-Mouse AF488 (ab150117, Abcam), Mouse anti-Lamin A (ab8980, Abcam), Mouse anti-Coilin (ab87913, Abcam), Mouse anti-SMN (ab5831, Abcam).

### Protein-RNA co-immunoprecipitation, RNA purification and RT-qPCR

Formaldehyde RNA immunoprecipitation (FRIP) of candidate BC200 interacting proteins was performed as previously described (46). The published protocol was modified slightly as follows: following lysis, sonication was performed three times for 10 s at 30% output and following crosslink reversal, RNA was purified using the GeneJet RNA cleanup and concentration micro Kit. Native RNA immunoprecipitations were performed by scraping the cells of one 150 mm dish of MCF-7 cells per IP into cold PBS. Cells were lysed as described above and immunoprecipitations were performed by combining 10 μg of antibody with 500 ul cell lysate (5 mg/ml in IP buffer) for 1 h at 4°C with end-over-end mixing. After antibody incubation, 50 μl pre-equilibrated protein A/G beads were added to the lysate and incubation continued for an additional hour. Following incubation beads were washed four-fold in IP buffer and 10% of sample was set aside for western blot. The remaining beads were heated at 95°C in 300 μl resuspension buffer of the RNA cleanup and concentration micro kit (Thermo-Scientific). Beads were pelleted and RNA was purified from the supernatant according to the manufacturer's protocol. For both native and crosslinking immunoprecipitations, RT-qPCR analysis was performed using an Applied Biosystems StepOnePlus instrument with the RNA to Ct One-step RT-qPCR kit (Thermo-Fisher Scientific). Reverse transcription and cycling parameters were carried out as per the manufacturer's specifications (Thermo-Fisher Scientific). To calculate percent input, RNA was similarly extracted from 10% of the total cell lysate used for IP to serve as a reference sample. Twenty five nanogram of template RNA was used in all RT-qPCR reactions. Reaction specificity was confirmed by melt-curve analysis as well as agarose gel electrophoresis of reaction products. A minimum of three independent experiments were performed for each sample and measured in triplicate. The following primers were used: BC200-forward, ATAGCTTGAGCCCAGGAGTT; BC200-reverse, GCTTTGAGGGAAGTTACGCTTAT; GAPDH-forward, ACCCACTCCTCCACCTTTG; GAPDH-reverse, CTCTTGTGCTCTTGCTGGG; CSDE1-Forward, AGACCGACGTGACAAATTAGAG; CSDE1-Reverse, GCAGCAATCACACCCATTTC; BC1-Forward1, GGGATTTAGCTCAGTGGTAGAG; BC1-Reverse1, AGGTTGTGTGTGCCAGTTA; BC1-Foward2, CGGTCCTCAGCTCTGGAAA; BC1-Reverse2, GTGTGTGCCAGTTACCTTGTT; 7SL-Forward, GCACTAAGTTCGGCATCAATATG; 7SL-Reverse, CTGATCAGCACGGGAGTTT.

### Recombinant protein expression, purification and electrophoretic mobility assays (EMSAs)

The cDNA for CSDE1 isoform 1 (NM_001007553) with an N-terminal FLAG tag was amplified from purified RNA extracted from MCF-7 cells and cloned and sequenced in the pCDNA3.1 vector using standard molecular biology techniques. The cDNA for STRAP with an N-terminal FLAG tag in the pCDNA3.1 vector was purchased from Genscript. Protein expression and purification were carried out as previously described (47). Binding reactions were performed in PBS buffer with 50 nM RNA and serial dilutions of protein from 1 μM to 7.8 nM. EMSAs were performed as described previously (14).

### siRNA, LNA GapmeR and plasmid transfection

siRNAs and LNA GapmeRs were transfected using Lipofectamine RNAiMax (Thermo-Fisher Scientific) according to the manufacturer's protocol. siRNAs were ordered from Thermo-Fisher Scientific (Silencer Select). The siRNA sequences are as follows: CSDE1_1: GCCUAAUGGUUCUUCGUCATT, CSDE1_2: GGUUGAAUUUAGUAUUAGUTT, STRAP_1: GGAUCAUGCUACUAUGACATT, STRAP_2: GCAUCACGCCUUAUGGGUATT. LNA GapmeRs were ordered from Exiqon (Woburn, MA). The BC200 targeting GapmeR sequence is as follows: AGGGAAGTTACGCTTA (Design ID# 569710–2). The Negative Control GapmeR sequence is as follows: AACACGTCTATACGC.

### BC200 Half-Life measurements

To assess the impact of CSDE1 on BC200 stability, cells were treated with Actinomycin D (5 μg/ml) to inhibit RNA polymerase III (48). Following treatment, an equal number of cells were harvested as per the indicated time points through 12 h. RNA was extracted using the GeneJet RNA purification kit and global RNA degradation was assessed by plotting the total RNA recovered from an equal number of cells at each time point. BC200 and 7SL levels were measured by RT-qPCR as described above on three biological replicates per time point. Decay rate constant (*K*) was calculated by fitting the data to the following one phase decay equation with GraphPad Prism 5: $y\ = ( {Y_0 - {\rm Plateau}} )\times \exp ( { - KX} ) + {\rm Plateau}$, where Plateau is equal to *Y* at infinite time. Half-life (*λ*) was calculated as $\lambda \ = \ln ( 2 )/K$.

### Immunofluorescence

For immunofluorescence experiments cells were grown on #1.5 glass coverslips in a 24-well dish. Fixation was performed with 4% formaldehyde in PBS for 10 min at room temperature, following which cells were washed threefold in PBS. Cells were permeabilized with 0.1% Triton-X 100 in PBS for 10 min at room temperature and washed three times for 5 min in PBS. Blocking was performed for 30 min in PBS containing 1% BSA and 22.5 mg/ml glycine followed by an additional 30-minute blocking step in 10% goat serum. Cover slips were incubated overnight in primary antibody in PBS containing 1% BSA at 4°C. The following antibodies were used: Anti-CSDE1 (HPA052221, Sigma-Aldrich) diluted 1:50; Anti-Coilin (ab87913, Abcam) diluted 1:100; Anti-Lamin A (ab8980, Abcam), diluted 1:100, Anti-SMN (ab5831, Abcam), diluted 1:300. Following incubation, cover slips were washed four times for 5 min in PBS and secondary antibodies were added at a 1:300 dilution in PBS with 1% BSA (Goat anti-Mouse IGG Alexa Fluor 488 conjugate, ab150117, Abcam, Goat anti-Rabbit IGG Alexa Fluor 647 conjugate, 111-605-003, Jackson Immunologicals). Incubation was performed for 1 h at room temperature and cover slips were washed four times for 10 min in PBS and mounted to glass slides with Prolong Diamond mounting media containing DAPI (Thermo-Fisher Scientific). Cells were imaged with a 100× oil immersion objective on an EVOS FL Auto imaging system (Thermo-Fisher Scientific).

## RESULTS

### Identification of BC200 binding proteins by mass spectrometry

In an effort to identify a comprehensive set of BC200 interacting proteins, BC200 was 3′ digoxigenin (DIG) labelled and transfected into HEK-293T, MCF-7, MDA-MB-231 and MEF cells. A DIG label was employed as it avoids unintended cellular interactions that a biotin tag would confer (49). Labeling efficiencies of all RNAs employed in this study were assessed by electrophoretic mobility shift assay (EMSA) using an anti-DIG antibody (Supplementary Figure S1A). As an alternative to incubating the labelled RNA in cell lysate, the RNA was transfected into cells for 24 h prior to lysis to allow the exogenous RNA to be incorporated into more biologically relevant complexes within the cell. The MCF-7 cell line was chosen as it expresses high levels of BC200 and shows a dramatic loss of cell viability and induction of apoptosis upon BC200 knock-down. MDA-MB-231 cells express moderate levels of BC200 and demonstrate growth arrest but not induction of apoptosis upon BC200 knockdown and additionally, HEK293T were employed as they exhibit minimal loss in viability upon BC200 knock-down (1). In order to identify non-specific RNA-protein interactions, an RNA was generated by scrambling the BC200 sequence (BCSCR) for comparative purposes. Finally, as the murine RNA BC1 is postulated to perform an analogous function in mice to the primate-specific BC200, parallel experiments were performed with the DIG-labelled BC1 RNA to compare and contrast the potential cellular functions of these RNAs. The sequences of all three labelled RNAs are shown in Supplementary Figure S1B.

**Figure 1.**
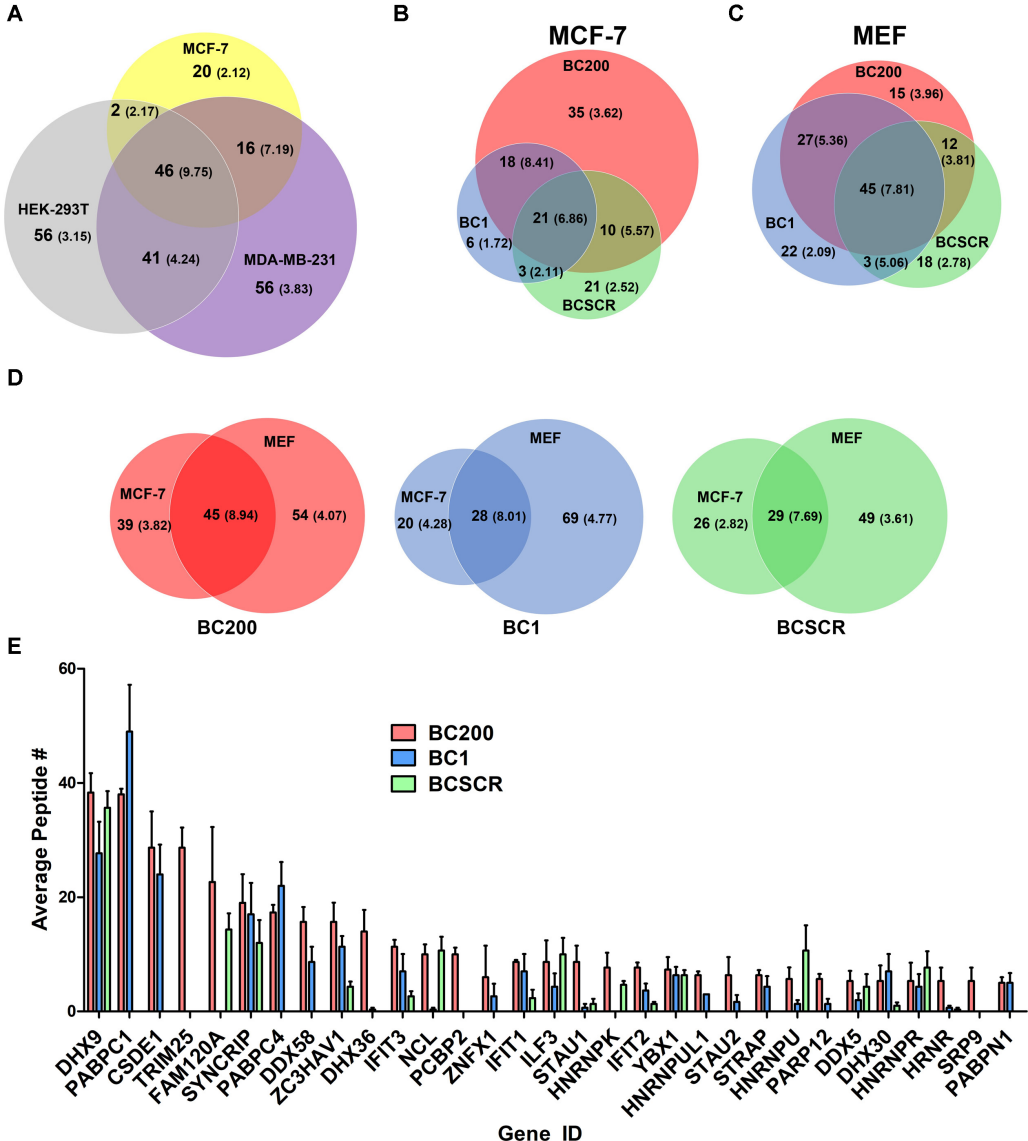
MS analysis of the BC200 RNP. (**A**) Venn diagram displaying the summary statistics of the MS analysis of the BC200 RNP in MCF-7, HEK-293T and MDA-MB-231 cell lines. Total number of proteins identified in each category are indicated by bolded numbers and average unique peptide numbers are reported in parentheses. (**B**) As in (A), summary statistics of the proteins bound to BC200, BC1 and BCSCR in MCF-7 cells. (**C**) As in (B) for MEF cells. (**D**) Comparison of shared and unique binding partners for BC200, BC1 and BCSCR between MCF-7 and MEF cells. (**E**) Average peptide numbers reported as a bar graph for the most abundant BC200 interacting proteins identified in MCF-7 cells compared to data obtained with the BC1 and BCSCR RNAs. Data represents the mean of three independent replicates ± standard deviation.

The proteins bound to BC200, BCSCR and BC1 were identified by on-bead trypsin digestion followed by nano-RP-LC-MS/MS. Three independent biological replicates were performed for each condition and the average number of unique peptides identified was used to compare results for each protein between cell lines and the various RNAs. All raw and filtered data is available in Supplementary File 1. In Figure 1A, the BC200-bound proteins identified in MCF-7, HEK-293T and MDA-MB-231 cells are compared as a Venn diagram with the number of unique proteins identified in each category followed by the average number of unique peptides for those proteins in parentheses. The 46 proteins common to all three cell lines demonstrated the highest average peptide number. Gene ontology analysis of all subsets was performed (Panther Overrepresentation Test of Biological Process) (50). The complete set of data is available in Supplementary File 2. A clear enrichment of proteins involved in RNA stability, folding, localization, splicing, and translation was observed in all three cell lines. BC200 complexes from HEK293T cells contained several proteins involved in nucleobase transport that were absent from MCF-7 and MDA-MB-231, whereas MDA-MB-231 BC200 complexes contained proteins involved in splicing reactions that were absent from the other two cell types. Unique proteins identified in MCF-7 cells were involved in innate immune response and viral defense, a possible artifact of exogenous RNA transfection as many of these proteins were also found bound to the scrambled RNA.

Analysis of proteins bound to BC200, BC1 and BCSCR in MCF-7 cells demonstrated that the majority of BC1 bound proteins also bind BC200 (Figure 1B). Similar results were observed in MEF cells; however, in this case a greater number of BC1 unique proteins were identified and a reduced number of BC200 specific proteins was observed (Figure 1C). Comparison between human and murine cells revealed a general trend in that the more abundant proteins (higher number of unique peptides identified) were common binding partners for each RNA (Figure 1D).

Gene ontology analysis performed on the proteins bound to BC200, BC1 and BCSCR in MCF-7 and MEF cells revealed considerable functional overlap (Supplementary File 3). Comparison of the BC200, BC1 and BCSCR binding partners with the highest number of identified peptides determined that a significant fraction of the proteins binding BC200 and BC1 were also binding to the scrambled sequence (Figure 1E). Therefore, to gain a clearer understanding of BC200 function, we pursued further experiments to stringently validate the binding partners. For these experiments we chose to focus on the MCF-7 cell line.

### Validation of BC200 bound proteins in MCF-7 cells

**Figure 2.**
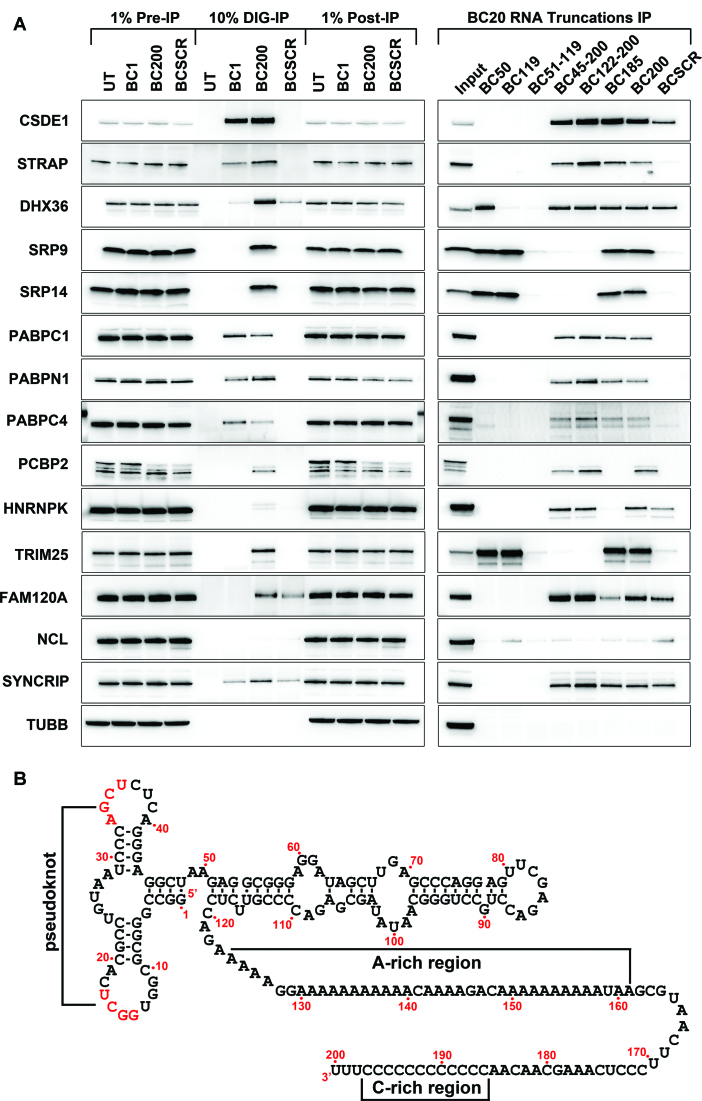
Western blot analysis of confirmed BC200 targets. (**A**) Panel 1: Western blots were performed with antibodies to the indicated proteins on pull-down samples of untransfected (UT, beads alone) and transfected BC1, BC200 and BCSCR RNAs. Panel 2: Western blots were performed as in Panel 1 on pull-down samples of the indicated DIG-labelled RNAs incubated in 500 μl cell lysate (5 mg/ml) at a concentration of 250 nM. (**B**) Schematic of the BC200 RNA demonstrating the predicted secondary structure.

To gain a clearer sense of the relative abundance and specificity of BC200 binding proteins in MCF-7 cells, western blots were performed on BC200, BC1 and BCSCR pull-down samples. Western blots were carried out with antibodies to 24 of the proteins identified by mass spectrometry, selected primarily based upon abundance ranking by average peptide number (Figure 2 and Supplementary Figure S2). To further assess the sequence requirements for each interaction, a pull-down was also performed with a series of six BC200 RNA truncations. Because several truncations demonstrated a reduction in cell viability upon transfection, DIG-labelled BC200 truncations were added to cell lysate rather than transfected. Three distinct modes of interaction were observed amongst the 14 confirmed binding partners, involving either the 5′ Alu domain (SRP9, SRP14, TRIM25), the 3′ A-rich region (CSDE1, STRAP, DHX36, PABPC1, PABPN1, PABPC4, SYNCRIP, FAM120A) or the 3′ C-rich region (PCBP2, HNRNPK) (Figure 2A, B).

**Figure 3.**
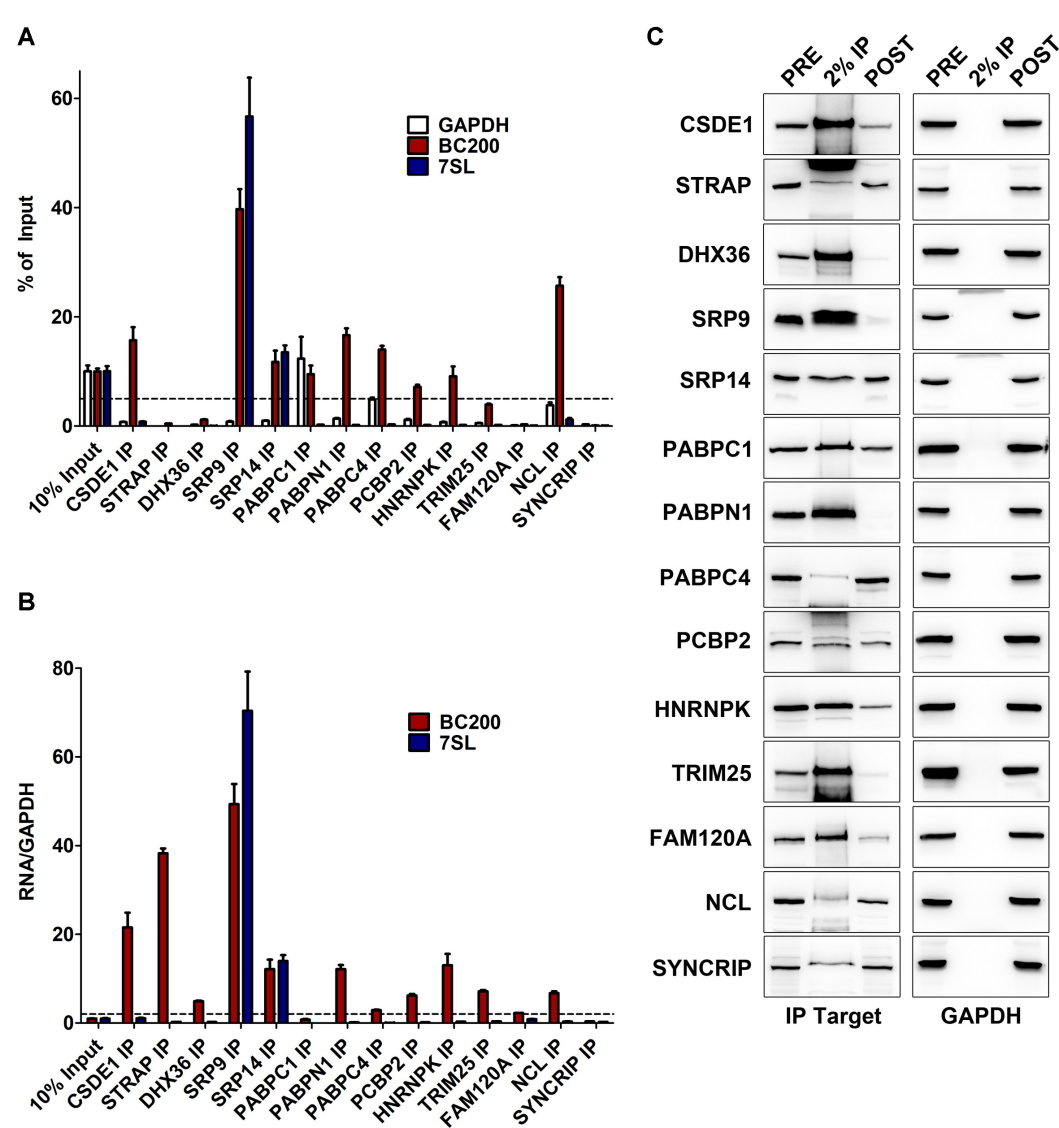
RNA Immunoprecipitation experiments of confirmed BC200 targets. (**A**) RT-qPCR analysis of BC200, GAPDH and 7SL enrichment by immunoprecipitation of the indicated proteins. RNA extracted from 10% of the input sample was used as a reference to calculate percent of input for each RNA that was bound to the immunoprecipitated protein. Data represents the mean of three independent replicates ± standard deviation. Dashed line represents the threshold value of 5% input. (**B**) Percent input values of BC200 and 7SL were compared to GAPDH to demonstrate the degree of specificity of the interactions analyzed in (A). Dashed line represents the threshold value of 2-fold enrichment. (**C**) Immunoprecipitation efficiency was monitored by performing western blot on 50 μg of PRE and POST IP samples as well as 2% of the IP.

**Figure 4.**
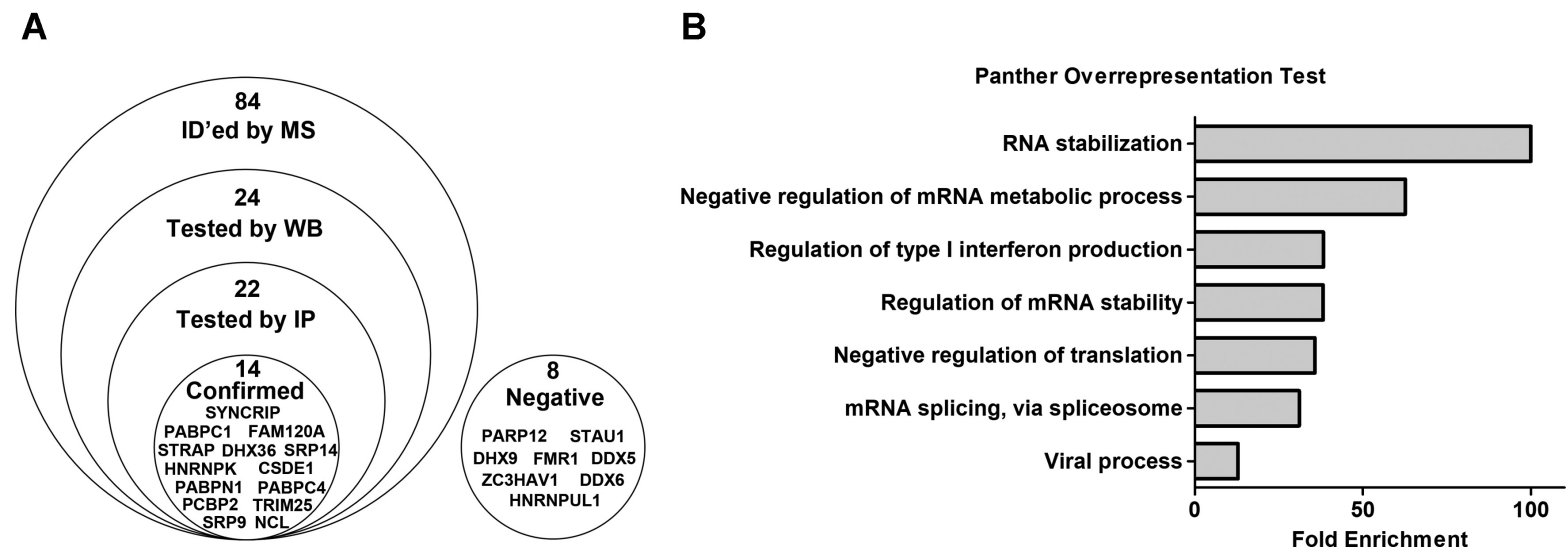
Protein Validation summary in MCF-7 cells. (**A**) Diagram representing the total proteins identified in MCF-7 cells (average peptide number > 1) and the progression of the validation process. (**B**) Panther overrepresentation test of the 14 confirmed BC200 binding partners as reported in (A).

To validate the interaction data obtained by BC200 IP, 22 of the identified proteins were immunoprecipitated from MCF-7 cells under both native and formaldehyde crosslinking conditions (Figure 3 and Supplementary Figure S3, S4). Immunoprecipitation efficiency was monitored by western blot (Figure 3C and Supplementary Figure S5). STAU2 and DDX58 were excluded from this validation step as STAU2 antibodies failed to IP and DDX58 was not expressed in untransfected MCF-7 cells. DDX58 IP in MDA-MB-231 cells that express basal levels of DDX58 failed to enrich BC200 (data not shown). RT-qPCR analysis of co-precipitating RNA was performed with primers specific for BC200 as well as two controls, GAPDH mRNA and the 7SL RNA. As % of input values are dependent upon protein immunoprecipitation efficiency, the ratio of bound BC200 to GAPDH was also plotted to demonstrate the specificity of the protein–BC200 interactions and correct for variations in IP efficiency (Figure 3B, Supplementary Figure S4). Proteins that either immunoprecipitated >5% of the input BC200 RNA or demonstrated a fold enrichment relative to GAPDH >2 were considered positive (dashed lines denote threshold values). These data led to the confirmation of 14 BC200 binding proteins and exclusion of eight proteins as likely non-specific interactions (Figure 4A).

Immunoprecipitation of the 14 validated proteins coupled with RT-qPCR analysis of the coprecipitating RNA demonstrated that the majority of the interacting proteins are bound to a substantial fraction of the cellular BC200 (Figure 3, Supplementary Figure S3). This is exemplified under native conditions where SRP9 co-precipitates ∼75% of the input BC200 RNA, a likely underestimation due to IP inefficiency and RNA loss through washing and purification steps (Supplementary Figure S3A). Despite incomplete IP, the novel binding partners CSDE1 and STRAP also bind to a significant fraction of the input BC200 (30% and 7% respectively) and exhibit a high degree of specificity relative to GAPDH and the 7SL RNA (Supplementary Figure S3 and S4).

While native immunoprecipitation conditions generally exhibited higher enrichment of BC200 largely due to reduced GAPDH co-immunoprecipitation, several interactions were dependent upon formaldehyde crosslinking prior to cell lysis (NCL, TRIM25, FAM120A, Supplementary Figure S3, S4). In contrast, SYNCRIP demonstrated enrichment of BC200 relative to GAPDH only under native conditions (Supplementary Figure S4). Gene ontology analysis exhibited enrichment of biological processes involved in RNA stability, metabolism, processing, splicing and translation (Figure 4B, Supplementary File 2). While these general processes give some direction in pursuing BC200 function, extensive analysis of the impact of these RNA-protein complexes on cell physiology is critical to understanding their role in the context of the BC200 RNP.

### CSDE1 binds directly to BC200 whereas STRAP interactions are CSDE1 dependent

**Figure 5.**
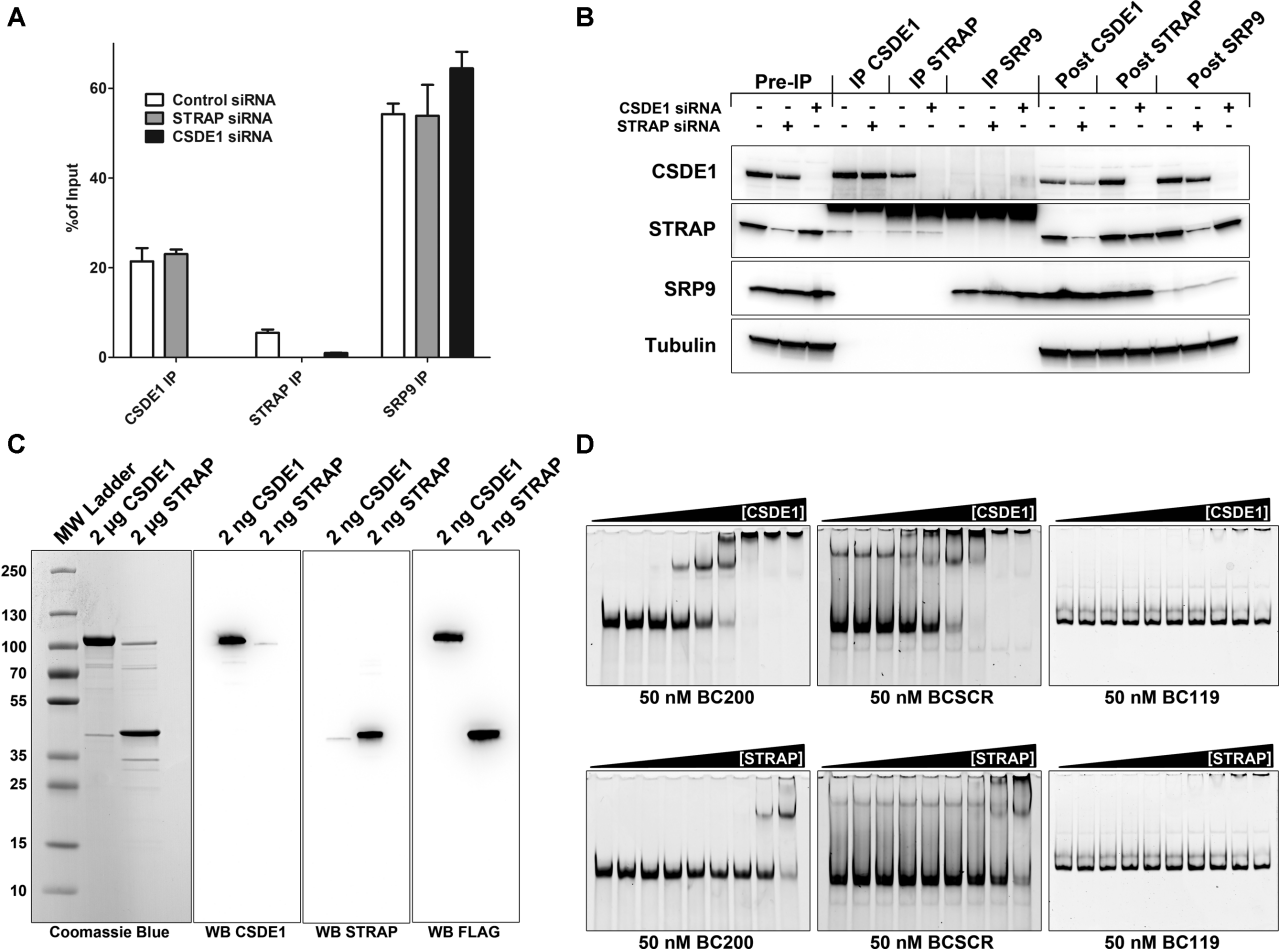
CSDE1 can bind directly to BC200 whereas STRAP interactions are CSDE1 dependent. (**A**) Immunoprecipitation experiments performed as described for Figure 3A under conditions of CSDE1 and STRAP knock-down by siRNA (48 h post transfection, CSDE1 siRNA_2, STRAP siRNA_2). Co-immunoprecipitating BC200 RNA was detected by RT-qPCR and compared to total RNA extracted from 10% of input. Data represents the mean of three independent replicates ± standard deviation. (**B**) Immunoprecipitation efficiency was monitored as in Figure 3C. (**C**) Panel 1: Coomassie stain gel of purified CSDE1 and STRAP separated by SDS/PAGE. Panel 2: Western blot with antibodies against CSDE1 of 2 ng purified CSDE1 and STRAP protein separated by SDS/PAGE. Panel 3: As in Panel 1, with antibodies to STRAP. Panel 4: As in Panel 1, with antibodies to FLAG peptide. (**D**) Electrophoretic mobility shift assays of binding reactions prepared with 50 nM BC200, BCSCR or BC119 and a concentration gradient of the indicated proteins. Serial dilutions of protein were used from 1000 to 7.8 nM. Gels were stained with SYBR Gold nucleic acid stain.

CSDE1 is a known single-stranded RNA binding protein that coordinates post-transcriptional regulation of genes involved in melanoma metastasis (27). STRAP on the other hand is a reported CSDE1 interacting protein that does not exhibit direct affinity for nucleic acids (29). To determine if the CSDE1 and STRAP interactions with BC200 are co-dependent, we performed immunoprecipitation of CSDE1 under conditions of STRAP siRNA knock-down in MCF-7 cells. In parallel we also performed STRAP immunoprecipitation under conditions of CSDE1 knock-down. Co-immunoprecipitation of BC200 under these conditions was assessed by purifying protein-bound RNA followed by RT-qPCR (Figure 5A). While STRAP siRNA had a negligible impact on BC200 enrichment by CSDE1, CSDE1 knock-down reduced STRAP binding by >80%. As a further control, SRP9 was immunoprecipitated in parallel under all conditions to demonstrate that CSDE1 knockdown specifically interferes with the STRAP-BC200 interaction. Neither CSDE1 or STRAP siRNA had a discernible impact on BC200 enrichment by SRP9 IP. IP efficiency was monitored by western blot of Input, IP and Post-IP samples (Figure 5B). Similar results were obtained with a second set of siRNAs targeting both CSDE1 and STRAP (Supplementary Figure S6A, B).

To further investigate the RNA-Protein interactions, we purified recombinant FLAG tagged CSDE1 and STRAP from HEK293T cells (Figure 5C). Proteins were of high purity; however, purified overexpressed STRAP and CSDE1 carried their endogenous heterodimerization partners with them in the final elution (Figure 5C, panels 1–3). Electrophoretic mobility shift assays were performed with BC200, BCSCR and BC119 (50 nM) and a range of protein concentration (7.8–1000 nM) for both CSDE1 and STRAP. While CSDE1 shifted the majority of free BC200 at a concentration of 125 nM, the same shift was not evident with STRAP until the highest protein concentration of 1000 nM. This shift is likely due to contamination of the purified protein with endogenous CSDE1. *In vitro*, CSDE1 did not display a preference for BC200 over the scrambled RNA BCSCR; however, no affinity was observed for the BC200 truncation BC119, which lacks the 3′ predominantly single stranded region of the RNA. CSDE1 also binds the 7SL RNA *in-vitro* (Supplementary Figure S6C) despite no evidence of an in-cell interaction (Figure 3). This confirms a general affinity of CSDE1 for single-stranded RNA, indicating that the specificity of the in-cell interaction is likely guided by additional binding partners. Supporting this notion, we did not observe CSDE1 affinity for BCSCR with the transfected RNA, but an interaction was observed when the RNA was supplemented to cell lysate (Figure 2A). These results highlight the importance of validating RNA-protein interactions within a cellular context.

To validate the CSDE1 interaction with murine BC1, we also performed immunoprecipitation of CSDE1 from MEF cells in a similar manner as described above. CSDE1 IP substantially enriched murine BC1, suggesting that both RNAs are likely involved in a similar cellular function involving CSDE1 (Supplementary Figure S7).

### CSDE1 and BC200 expression levels are mutually dependent

**Figure 6.**
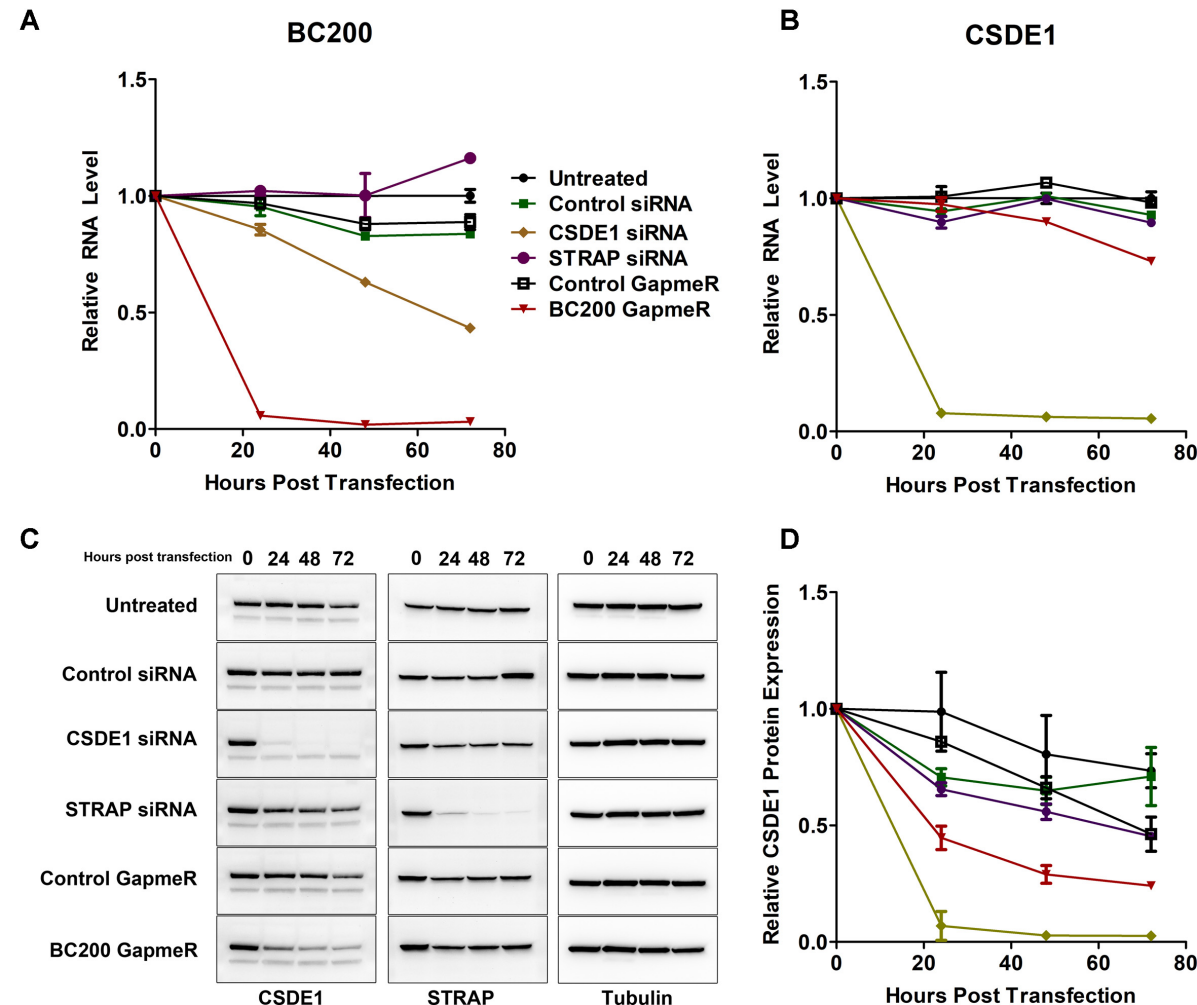
BC200 and CSDE1 expression are mutually codependent. (**A**) RT-qPCR analysis of BC200 RNA expression following transfection with the indicated RNA interference oligonucleotides. Data represents the mean of three independent replicates ± standard deviation. (**B**) RT-qPCR analysis of CSDE1 mRNA expression following transfection with the indicated RNA interference oligonucleotides. Data represents the mean of three independent replicates ± standard deviation. (**C**) Western blot analysis of protein samples from a 72-hour knock-down time-course with the indicated RNA interference oligonucleotides and antibodies. Data is representative of three independent replicates. (**D**) Densitometry measurements of CSDE1 protein expression from (C) as well as replicate experiments. Data represents the mean of three independent replicates ± standard deviation

To begin to explore the functional relationship between CSDE1 and BC200 we initially performed RNA interference-based knock-down experiments. Knock-down of CSDE1, but not STRAP, caused a steady reduction in BC200 RNA levels to ∼50% by 72 h (Figure 6A). Similar results were observed with additional siRNAs targeting both genes and neither CSDE1 nor STRAP siRNA impacted expression of GAPDH or the 7SL RNA (Supplementary Figure S8A, B). On the other hand, BC200 knock-down had minimal impact on CSDE1 mRNA expression at early time points but caused a significant reduction by 72 h to ∼75% (Figure 6B). In contrast to the CSDE1 mRNA level, CSDE1 protein expression was reduced at 24 h to 45% and was further reduced by 72 h to ∼20% of the 0 h time point (Figure 6C, D). As CSDE1 expression is markedly higher in G2/M-phase, a reduction of CSDE1 expression was observed at later timepoints under all treatment conditions due to cell confluence (51). As the protein levels of CSDE1 were reduced rapidly without significant change at the mRNA level, altered CSDE1 expression in the context of BC200 knock-down is likely due to a change in mRNA translation rate or protein stability.

### CSDE1 knock-down destabilizes BC200

**Figure 7.**
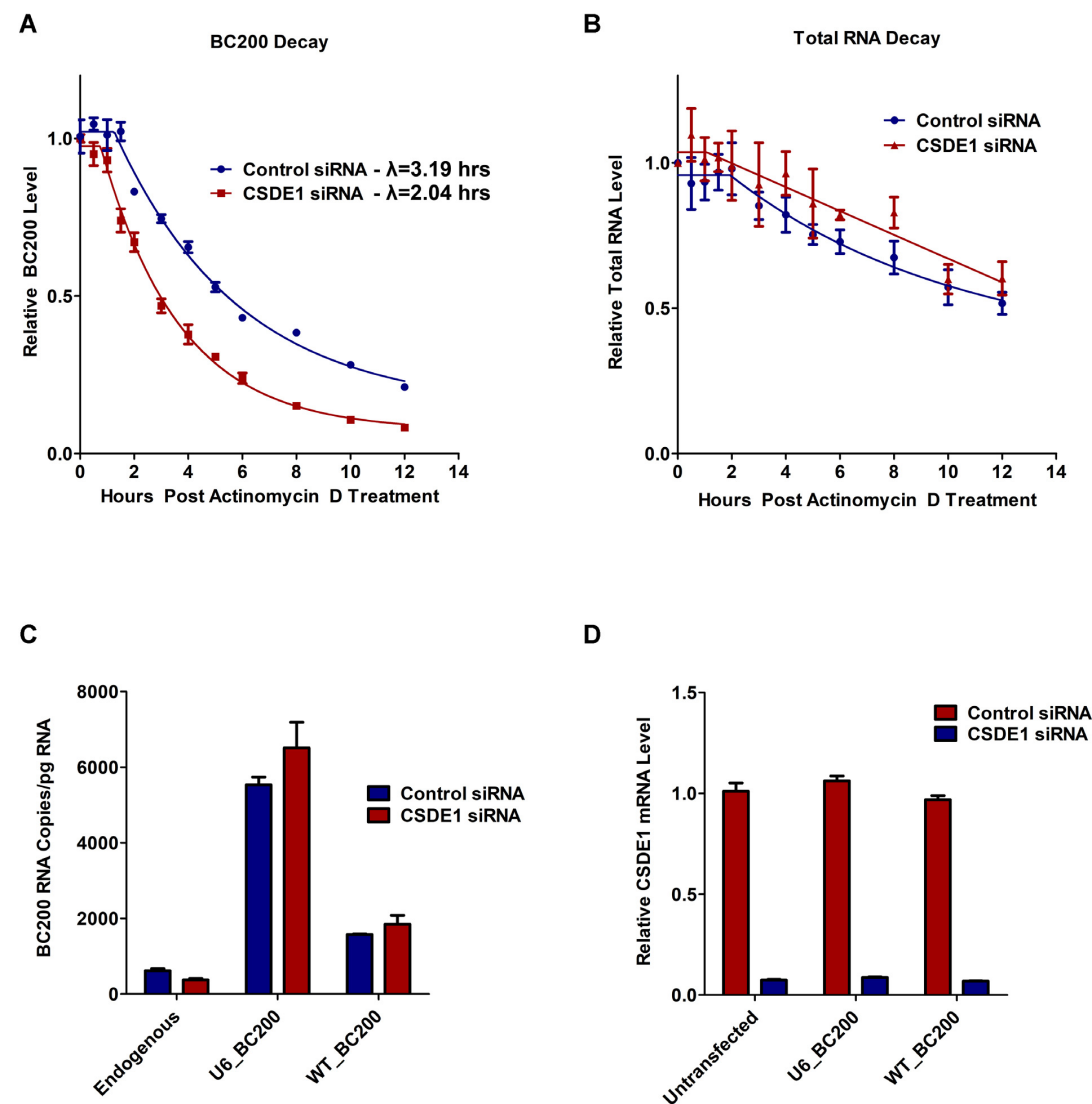
CSDE1 knock-down decreases stability of the BC200 RNA. (**A**) RT-qPCR analysis of BC200 expression following 72-hour CSDE1 or control knock-down and Actinomycin D treatment at *T* = 0. Indicated half-lives were calculated by fitting the data to a one-phase decay equation with GraphPad Prism software. Data represents the mean of three independent biological replicates measured in duplicate. (**B**) Relative total RNA quantities purified at the indicated time points from an equal number of cells to monitor total RNA decay. Data represents the mean of three independent replicates ± standard deviation. (**C**) MCF-7 cells were reverse transfected with control or CSDE1 siRNA and following 24 h were forward transfected with the plasmids containing BC200 under control of either the U6 snRNA promoter or endogenous BC200 promoter. Absolute expression levels of RNA from plasmid was calculated by RT-qPCR in parallel to a standard curve generated with purified BC200 RNA. Data represents the mean of three independent replicates ± standard deviation. (**D**) Relative CSDE1 mRNA expression was monitored by RT-qPCR analysis of the same RNA samples as used in (C).

To assess if the reduced steady state level of BC200 upon CSDE1 knock-down was a consequence of a change in the rate of BC200 decay, we knocked down CSDE1 for 72 h and then inhibited global transcription by treatment of the cells with 5 μg/ml Actinomycin D (48). An equal number of cells were harvested at each of the indicated timepoints and BC200 levels were quantified by RT-qPCR (Figure 7A). Decay rates under control and CSDE1 knock-down conditions were calculated by fitting the data to an equation modeling one-phase decay. The half-lives determined demonstrated a 40% reduction in the half-life of BC200 under CSDE1 knock-down. While a significant change was observed for BC200, global RNA decay was unchanged as was observed by plotting the relative change in total RNA purified at each timepoint (Figure 7B). A second CSDE1 siRNA exhibited a similar impact on BC200 decay and CSDE1 knockdown did not significantly impact decay of the 7SL RNA (Supplementary Figure S8C, D). Half-life measurements in MCF-7 cells are in good agreement with a previously published study (52).

To test if activity of the BC200 promoter was also altered, we introduced plasmids containing BC200 under control of the U6 snRNA promoter or within an endogenous genomic context (–2314 through +1474 of the BC200 gene). If transcription of the BC200 gene were specifically reduced, we would expect to see less BC200 expressed from the endogenous promoter vector under CSDE1 knock-down as compared to the control whereas the U6 snRNA promoter would only be impacted by post-transcriptional BC200 regulation. In contrast to this, we observed a similar trend, with a modest increase in BC200 levels generated from the reporter constructs under conditions of CSDE1 knock-down (Figure 7C, D). Therefore, we conclude that the reduced BC200 expression observed upon CSDE1 knock-down is primarily a result of decreased stability of the RNA. The stability of transfected BC200 RNA from both promoters was not impacted by CSDE1 siRNA, a result consistent with the observations of Kim *et al.* who report significantly reduced stability of plasmid transcribed BC200 as compared to the endogenous RNA (52).

### BC200 knock-down reduces CSDE1 cytoplasmic expression and reorganizes CSDE1 into distinct nuclear foci

SRP9/14, as part of the signal recognition particle, bind to newly translated signal peptides and delay protein translation until the target mRNA is localized to the ER membrane (53). As a substantial portion of the cellular BC200 is bound by SRP9/14 (Figure 3A) and as we had evidence to suggest post transcriptional regulation of CSDE1 by BC200, we sought to assess whether BC200 knock-down had an impact on the cellular localization of CSDE1. We hypothesized that BC200 may be involved in a similar yet distinct cellular role of translational repression during mRNA trafficking.

**Figure 8.**
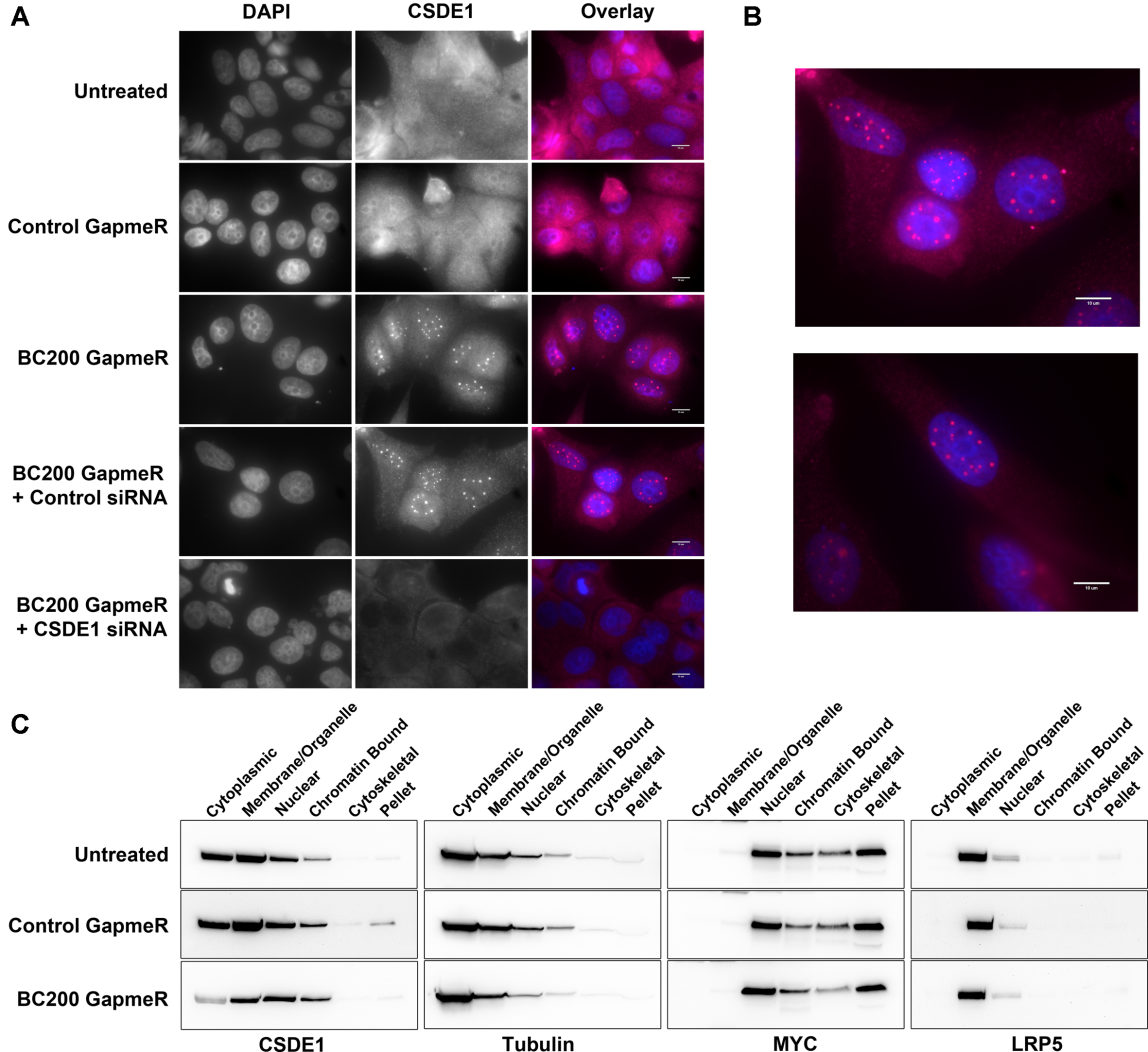
BC200 knock-down results in decreased cytoplasmic expression and localization of CSDE1 to concentrated nuclear foci. (**A**) Immunofluorescent analysis of MCF-7 cells transfected with the indicated RNA interference oligonucleotides. Cells were probed with anti-CSDE1 antibodies and counter-stained with DAPI. Scale bars indicate 10 μM. (**B**) Magnified representative images of CSDE1 localization following BC200 knock-down. (**C**) Western blot analysis of the subcellular distribution of CSDE1 following transfection of either control or BC200 targeting LNA GapmeR. Blots were subsequently probed with antibodies to Tubulin (cytoplasmic), MYC (Nuclear) and LRP5 (Membrane) to control for loading and fraction specificity.

CSDE1 localization was determined by immunocytochemistry under untreated conditions and 24 h after transfection with either the control or BC200 targeting LNA GapmeR. Under these conditions we observed a distinct reorganization of CSDE1 into highly concentrated discrete nuclear foci (Figure 8A, B). Subcellular fractionation revealed that, while BC200 knock-down dramatically reduced CSDE1 expression in the cytoplasmic and membrane/organelle fractions, nuclear CSDE1 remained largely unchanged (Figure 8C).

**Figure 9.**
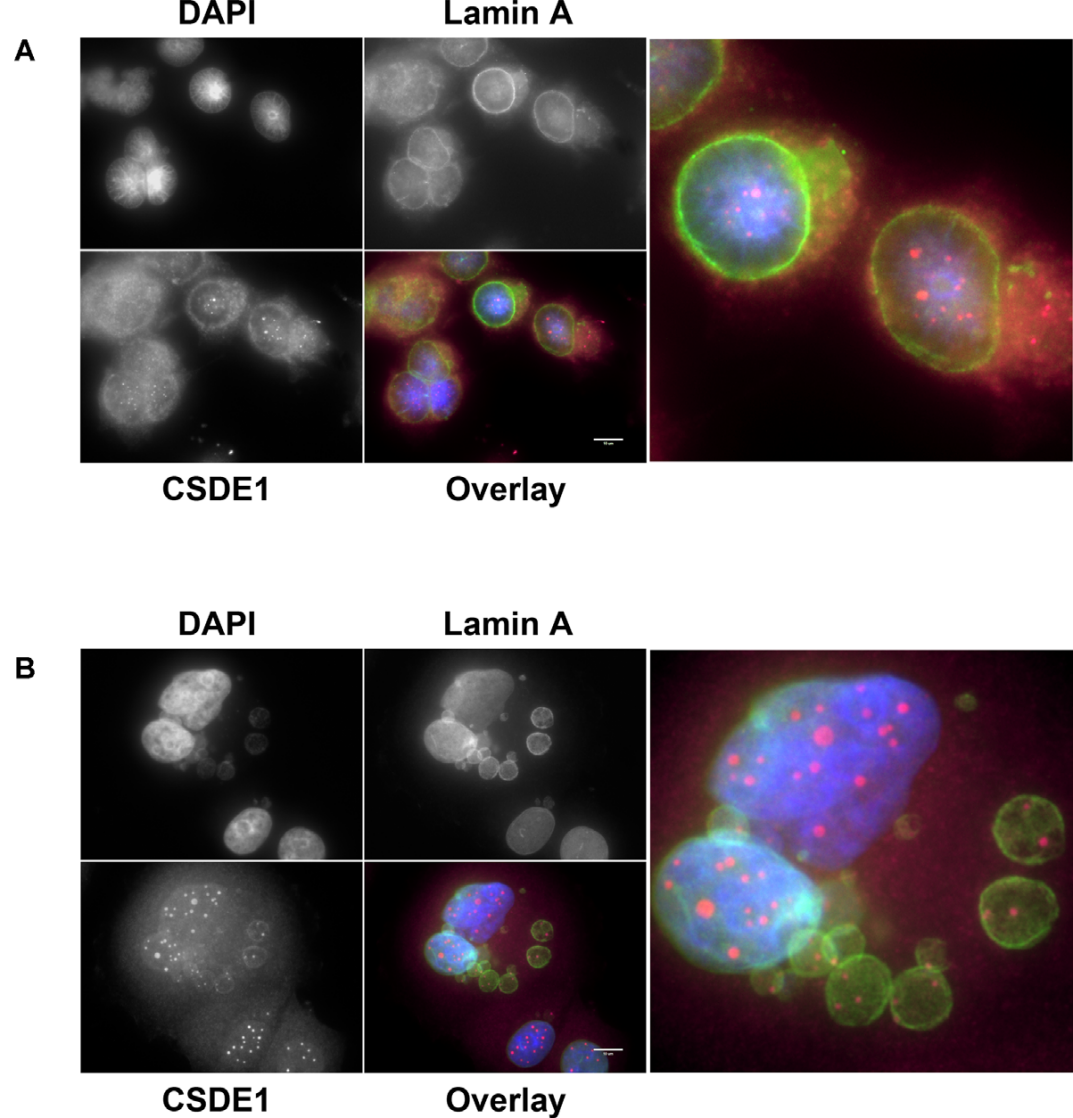
CSDE1-rich foci are not equivalent to previously described UNR-rich nucleoplasmic reticulum. (**A**) Immunofluorescent analysis of MCF-7 cells transfected with BC200 targeting GapmeR. Cells were probed with antibodies to CSDE1 (magenta) and Lamin A (green) and counter-stained with DAPI. (**B**) As in (A) highlighting nuclear localization in budded apoptotic nuclear membranes. Scale bars indicate 10 μM.

While CSDE1 expression is reported to be primarily cytoplasmic, a recent study described localization of CSDE1 within cytoplasmic invaginations into the nucleus (nucleoplasmic reticulum) under stress conditions and in normal polyploid cells (36). To determine if we were observing a similar phenomemon, we co-stained cells transfected with BC200 GapmeR with antibodies targeting CSDE1 and Lamin A (nuclear envelope marker). Contrary to the observations of Saltel *et al.*, we did not observe a distinct nuclear envelope surrounding the CSDE1-rich foci (Figure 9A) (36). Furthermore, in apoptotic cells, CSDE1-foci were constrained to the inside of budded nuclear envelope fragments, a hallmark of nuclear envelope breakdown during apoptosis (54) (Figure 9B). These data support the subcellular fractionation experiments performed which suggest that the CSDE1 signal observed is truly nuclear (Figure 8C).

**Figure 10.**
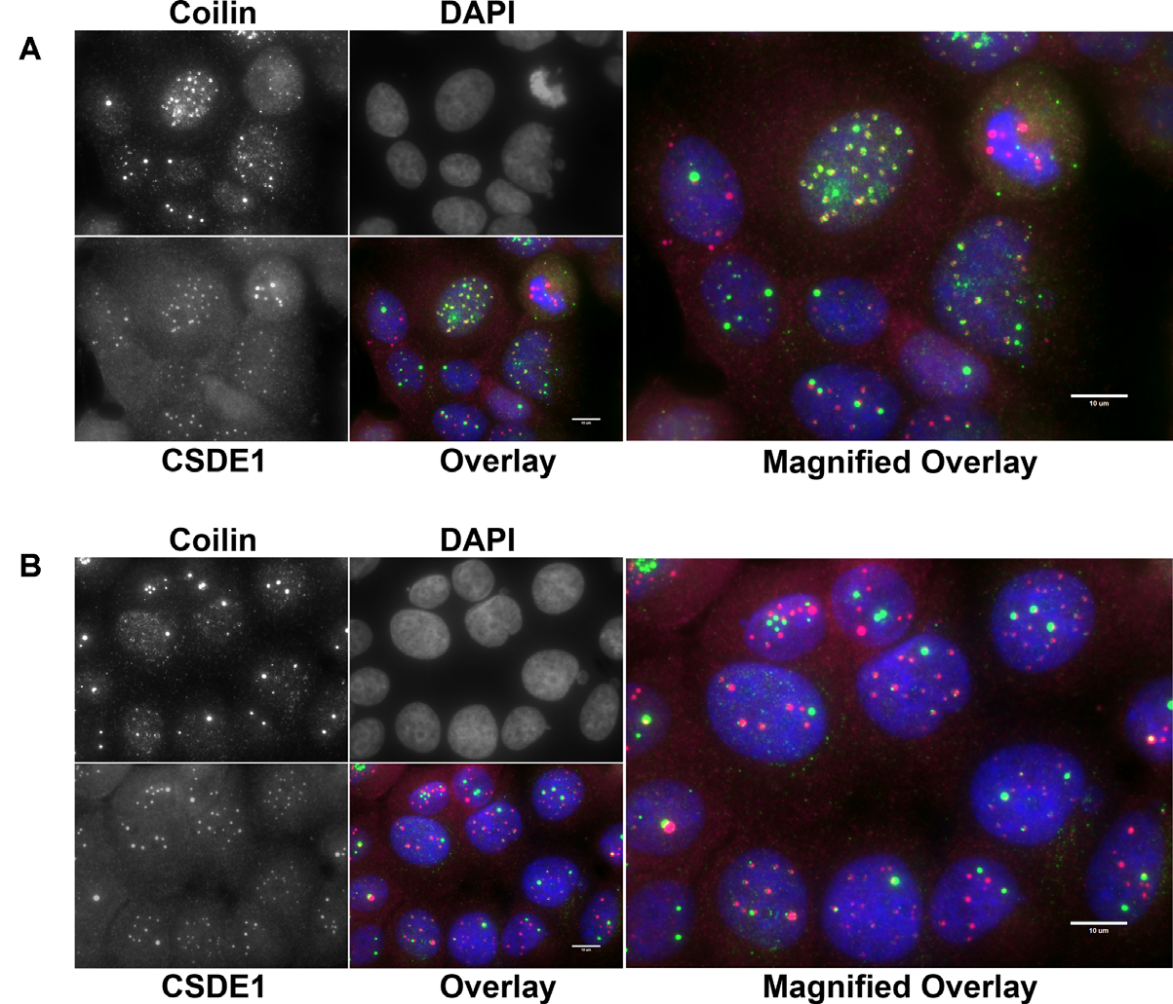
CSDE1-rich foci associate with coiled bodies in a subpopulation fixed cells. (**A**) Immunofluorescent analysis of MCF-7 cells transfected with BC200 targeting GapmeR. Cells were probed with antibodies to CSDE1 (magenta) and Coilin (green) and counter-stained with DAPI. Cell in centre of the field of view demonstrates complete colocalization of the nuclear foci. (**B**) As in (A) highlighting cells in which colocalization of foci is not evident. Scale bars indicate 10 μM.

**Figure 11.**
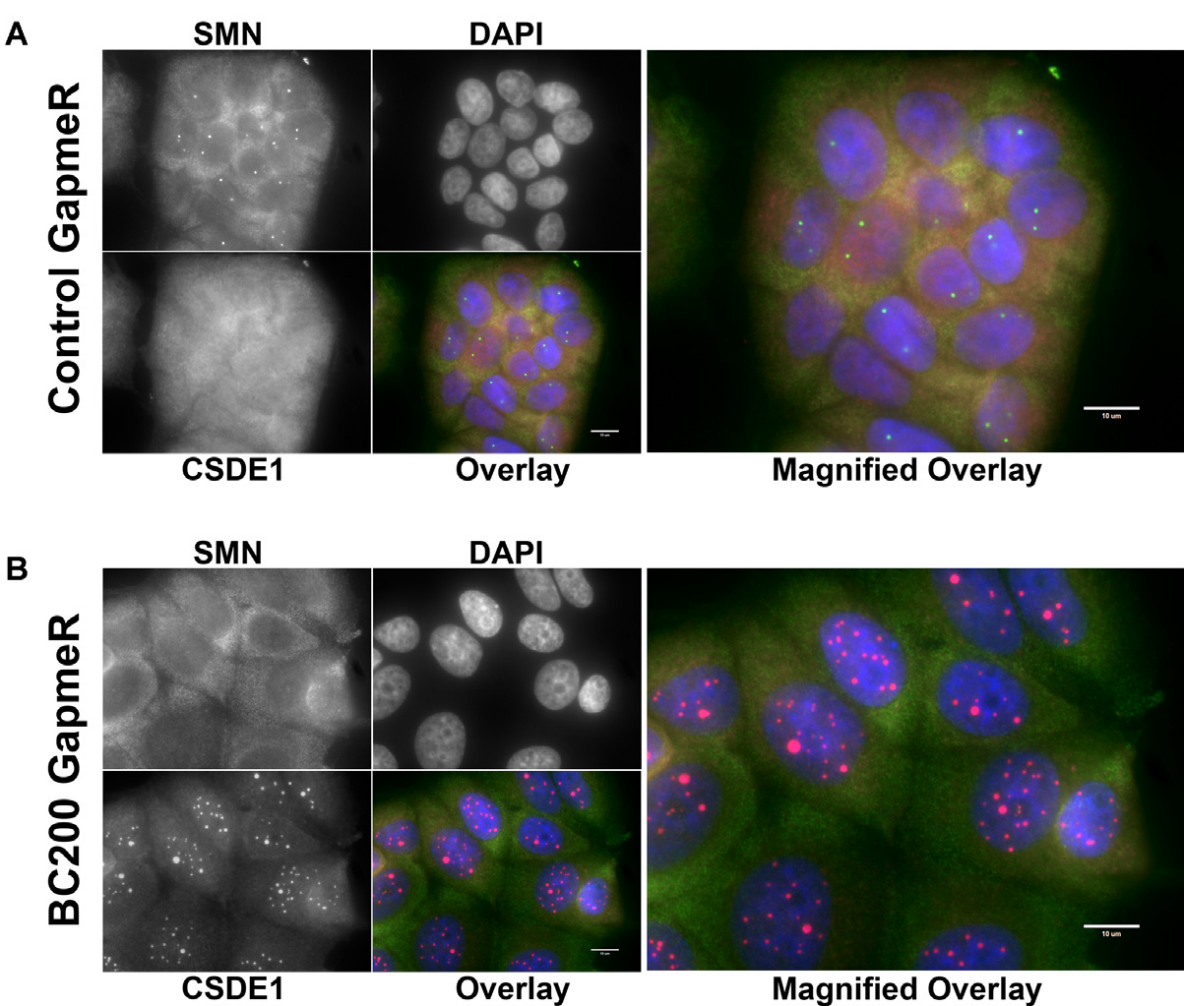
CSDE1-rich foci do not colocalize with nuclear gems. (**A**) Immunofluorescent analysis of MCF-7 cells transfected with control GapmeR. Cells were probed with antibodies to CSDE1 (magenta) and SMN (green) and counter-stained with DAPI. (**B**) As in (A) MCF-7 cells were transfected with BC200 targeting GapmeR causing reorganization of CSDE1 into nuclear foci and loss of nuclear SMN. Scale bars indicate 10 μM.

While CSDE1 has not previously been described to associate with subnuclear bodies, the CSDE1 interacting protein STRAP has been implicated in the assembly of cajal or coiled bodies, a sub-nuclear domain abundant in the protein Coilin and nuclear Gems, abundant in the SMN protein (55). STRAP binds to the survival of motor neuron (SMN) complex in a manner mutually exclusive of CSDE1 binding and siRNA knock-down of STRAP results in enhanced localization of SMN into nuclear gems and/or coiled bodies (37). In light of this, we co-stained MCF-7 cells with anti-CSDE1 and anti-Coilin antibodies to determine if CSDE1 is localizing to coiled bodies under BC200 knock-down. While a nearly perfect co-localization was observed in many cells (Figure 10A), the majority of cells exhibited staining that would suggest that CSDE1-rich foci are distinct from coiled bodies (Figure 10B). Mitotic cells demonstrated disassembly of coiled bodies whereas the CSDE1 rich foci remained intact (Supplementary Figure S9A); thus, the variation in association with coilin may be representative of a dynamic process in which CSDE1 foci are localized to coiled bodies at a particular stage in the cell cycle. Supporting this is the observation that cells with higher numbers of coiled bodies of small diameter, a hallmark of G1 phase, tended to show a high degree of colocalization whereas cells with fewer and larger coiled bodies, a hallmark of G2, did not (55,56). Thus, while CSDE1-rich nuclear foci do appear to have some relation to coiled bodies, the precise nature of these subnuclear domains is yet to be determined. Untreated cells, or cells transfected with the control GapmeR did not demonstrate any colocalization of CSDE1 and Coilin (Supplementary Figure S9B). Finally, we assessed whether CSDE1 nuclear foci co-localized with the survival of motor neuron protein (SMN), a marker of nuclear gems, a subnuclear domain closely associated with coiled bodies and involved in snRNP biogenesis (38). Under control conditions, SMN exhibits an expected pattern of cytoplasmic staining and localization to distinct nuclear domains; however, under conditions of BC200 knock-down the nuclear staining of SMN is completely abolished (Figure 11A, B). Therefore, the CSDE1-rich nuclear foci do not represent nuclear gems and in addition to CSDE1 reorganization within the nucleus BC200 knock-down results in either disassembly of nuclear gems or dissociation of SMN from these subnuclear domains.

## DISCUSSION

The importance of lncRNAs to cell biology in both normal and diseased states is becoming increasingly evident. lncRNAs have been implicated in nearly every facet of cellular physiology from epigenetic regulation, to modulation of gene transcription, mRNA translation, stability and splicing and as protein-co factors in a myriad of biological processes (57,58). In this study, we have focused on elucidating the function of the lncRNA BC200 through comprehensive proteomic analysis of binding partners followed through with stringent validation and exploration of the relationship between BC200 and the novel binding partner CSDE1. The function of BC200 is of particular interest in that several recent studies have demonstrated that this RNA is critical for tumour cell viability, cell migration and metastasis (1,7,15,16). While significant future work remains, this study sheds light onto BC200 function and provides a basis from which we anticipate future studies will further clarify the cellular role of this lncRNA.

Proteomic analysis of the BC200 RNP in several cell lines revealed a core set of abundant common binding partners as well as proteins that exhibited distinct interactions in a cell type dependent manner. Both BC200 and BC1 were bound to proteins involved in similar biological processes in human and murine cell lines; however, the interaction data of the scrambled RNA BCSCR demonstrated that stringent validation was necessary to separate true binding partners from experimental artifacts. As such, to keep the study feasible, we focused our efforts on a single breast tumour cell line, MCF-7, that exhibits a high degree of sensitivity to BC200 knock-down induced apoptosis. As peptide numbers are a crude means of estimating protein quantity, western blots were performed on 24 BC200 binding partners to gain a better sense of relative protein abundance. A series of BC200 truncations employed also revealed three distinct modes of interaction utilized by the 24 tested interacting proteins. These interactions were dependent upon either the 5′ Alu-domain, the 3′ A-rich or 3′ C-rich regions of BC200. As multiple sites of interaction are present on BC200, it is quite likely that many of these RNA-protein interactions are not mutually exclusive and several proteins may be present in a higher order complex.

While western blots were able to give a clearer indication of the specificity of each interaction, the ability of a protein to bind the BC200 scrambled sequence, BCSCR, does not necessarily preclude it from having a biologically relevant BC200 interaction. To probe this more stringently, reverse experiments were performed by immunoprecipitating the target proteins and assessing binding to endogenous BC200 by RT-qPCR. These experiments refined our starting list of 22 proteins down to 14 interactions that were biologically relevant and eight that were likely due to non-specific interaction. Amongst the eight excluded proteins was the previously reported binding partner, FMR1. Furthermore, SYNCRIP bound to only a small fraction of the input BC200 and demonstrated marginal enrichment. Our inability to demonstrate robust interactions with these proteins may be due to cell-type specificity of the interactions, as both FMR1 and SYNCRIP interactions were previously demonstrated in neuronal cell extracts (10,22,23). The data from these experiments demonstrated a trend in that proteins that exhibited significant binding to the scrambled RNA (DHX9, ZC3HAV1, DDX58, HNRNPUL1, DDX5, DDX6) were not confirmed BC200 interacting partners.

Amongst the 14 confirmed interactions, six of the proteins were previously described BC200 binding partners (PABPC1, DHX36, PCBP2, SRP9, SRP14, SYNCRIP) whereas eight of the proteins are novel BC200 interacting partners (PABPC4, HNRNPK, STRAP, PABPN1, CSDE1, TRIM25, FAM120A, NCL). It is quite possible that biologically relevant interactions exist amongst the 62 proteins not tested despite the relatively low number of unique peptides identified. This is exemplified by SRP9 and SRP14 for which the data support a clearly relevant interaction, but the MS screen yielded low peptide numbers, likely due to the low molecular weights of these two proteins. While several of the confirmed interactions were previously reported, reverse experiments by quantitative RT-qPCR shed light on the relative fraction of cellular BC200 bound by these and the newly identified proteins. Despite imperfect IP efficiency, percent of input calculations revealed that under native conditions SRP9 is bound to 75% of the total BC200 in the cell lysate. Comparing IP efficiencies to the percent input bound revealed that, with the exception of HNRNPK, DHX36, FAM120A and SYNCRIP, the majority of interacting partners bound to a substantial fraction of cellular BC200. While the exact nature and heterogeneity of the BC200 RNP remains to be resolved, it seems most probable that several of these interactions are occurring simultaneously.

As CSDE1 and STRAP exhibited a high degree of BC200 enrichment relative to GAPDH they were selected for further study. Consistent with previous findings, the STRAP interaction appears to be dependent on direct protein–RNA contacts made by its heterodimerization partner CSDE1. Interestingly, as measured by *in-vitro* binding reactions and in pull-downs where RNA was supplemented to cell lysate rather than transfected into the cells, CSDE1 did not demonstrate a specific affinity for BC200 relative to BCSCR. This strongly indicates that the specificity of the interaction is dependent upon additional interactions and/or subcellular localization that is only realized within a cellular context. While a direct interaction between CSDE1 and BC200 is demonstrated *in-vitro*, our data cannot rule out the possibility that the in-cell interaction is mediated by additional factors.

Knock-down studies of BC200 and CSDE1 revealed a mutual co-dependence of expression wherein CSDE1 expression stabilizes the BC200 RNA and CSDE1 post-transcriptional regulation is contingent upon BC200 expression. As CSDE1 has a reported function as an RNA chaperone (59), it is possible that CSDE1 facilitates the correct folding of BC200 and misfolded transcripts that accumulate in the absence of CSDE1 are degraded. An additional possibility is that CSDE1 binding to the exposed single stranded tail of BC200 prevents endonucleolytic cleavage of the RNA. While the impact of CSDE1 on BC200 half-life is quite clear, the mechanism by which CSDE1 protein levels are attenuated upon BC200 knockdown requires further study. CSDE1 expression is tightly regulated throughout the cell cycle, if BC200 knock-down is arresting cells outside of G2/M it would result in reduced protein expression possibly through an indirect mechanism. On the other hand, CSDE1 is known to autoregulate its expression via direct contacts within its 5 UTR (51), this process may be facilitated through a complex involving BC200. Further studies that can decipher whether BC200 acts directly at the CSDE1 mRNA should help to clarify this further.

In addition to attenuated expression, BC200 knock-down caused a dramatic redistribution of CSDE1 into highly concentrated nuclear foci. Follow up analysis revealed these to be distinct from the previously described UNR-rich nucleoplasmic reticulum (36) and co-localization studies demonstrated that while a subset of these nuclear bodies are likely associated with coiled or cajal bodies, the majority are devoid of Coilin protein. This raises the possibility that, in addition to regulating the post-transcriptional regulation of CSDE1, BC200 may be directly involved in dictating the subcellular distribution of the protein. Furthermore, BC200 knock-down was demonstrated to impact assembly of SMN into nuclear gems, a process previously reported to involve STRAP. Interaction of STRAP with Gemin-7 is reported to be mutually exclusive of its interaction with CSDE1, raising the possibility that BC200 mediated CSDE1 redistribution is impacting SMN complex assembly and localization via alteration of STRAP accessibility. The mechanisms undergirding the nuclear accumulation of CSDE1 into distinct foci and the loss of SMN within nuclear gems warrant further study. Future proteomic analysis of nuclear CSDE1 interactions within the context of BC200 knock-down should aid in deciphering the nature and function of these subnuclear domains.

In summary, we have detailed a comprehensive list of BC200 interacting proteins in multiple cell lines. Stringent validation has revealed eight novel interacting proteins and confirmed six previously reported interactions. Follow up studies with CSDE1 and STRAP validated the utility of the screen to uncover novel, relevant interactions and expose complex regulatory relationships. We expect this work will serve as the basis from which ourselves and others can continue to explore the molecular functions of this lncRNA and the protein binding partners involved in its function.

## Supplementary Material

Supplementary Data
