# Supplementary material for: Comprehensive analysis of the BC200 ribonucleoprotein reveals a reciprocal regulatory function with CSDE1/UNR
Source: Nucleic Acids Res. 2018 Sep 22;46(21):11575–91. doi: 10.1093/nar/gky860 (PMC6265466; doi:10.1093/nar/gky860)
Supplement: Supplementary Data [file gky860_supplemental_files.zip › Revised Supplementary Figures.pdf]

A

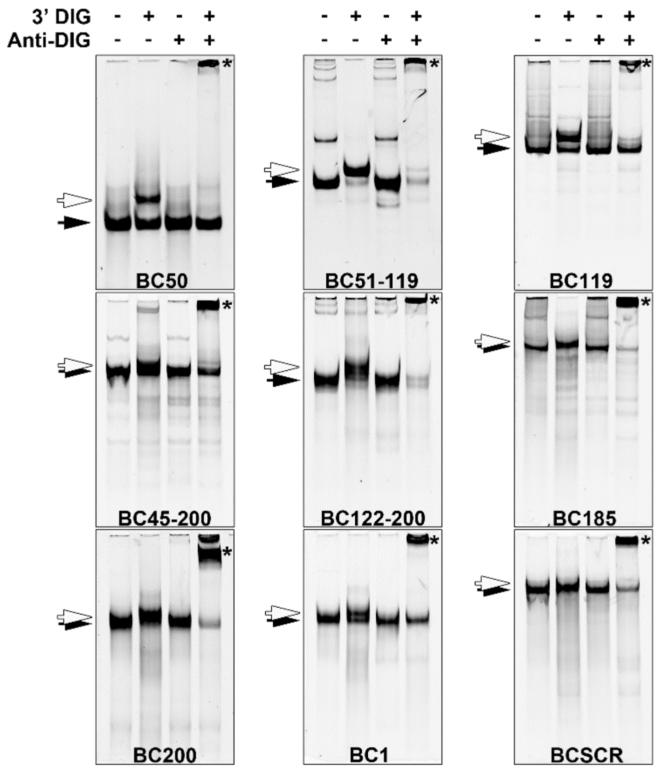

B

BC200

```
  1  GGCCGGGCGC GGUGGCUCAC GCCUGUAAUC CCAGCUCUCA GGGAGGCUAA GAGGCGGGAG
 61  GAUAGCUUGA GCCCAGGAGU UCGAGACCUG CCUGGGCAAU AUAGCGAGAC CCCGUUCUCC
121  AGAAAAAGGA AAAAAAAAAA CAAAAGACAA AAAAAAAAUA AGCGUAACUU CCCUCAAAGC
181  AACAACCCCC CCCCCCCUUU-ACGUA-DIG
```

BCSCR

```
  1  GGAUCAACAG CACAGGAUGU CUCAAACAGA GCGAUACCGG AGGACAUGGU CAACCCGCCA
 61  GCAGGACAUG CACGGCAAGC UCCUGAACGA GACCAUCACG ACAACCGCGA GUCAACGCCC
121  CAGUGAAUGA ACAGACCAGG GCCCAUACAA AGGAGAGGAU GGAAGAUAGA UGCCAUCCAC
181  UAAAUUCAUU GCCACCUUUU-ACGUA-DIG
```

BC1

```
  1  GGGGUUGGGG AUUUAGCUCA GUGGUAGAGC GCUUGCCUAG CAAGCGCAAG GCCCUGGGUU
 61  CGGUCCUCAG CUCUGGAAAA AAAAAAAAAA AAAAAAAAAG ACAAAAUAAC AAAAAGACCA
121  AAAAAAAACA AGGUAACUGG CACACACAAC CUUU-ACGUA-DIG
```

Supplementary Figure 1. RNA DIG-labelling efficiency and sequences employed. (A) DIG-labelled and unlabeled RNAs were incubated in binding reactions with a threefold molar excess of anti-DIG IgG. EMSAs were used to monitor DIG labelling efficiency of all RNAs used in the study. Gels were stained with SYBR gold. Solid arrows indicate unlabeled RNA, white arrows indicate DIG-labelled RNA and asterisk indicates RNA complexes with anti-DIG antibody. **(B)** Sequences of human BC200, a scrambled mutant of BC200 and the murine RNA BC1. Ligated DIG labeled linker is indicated in italics.

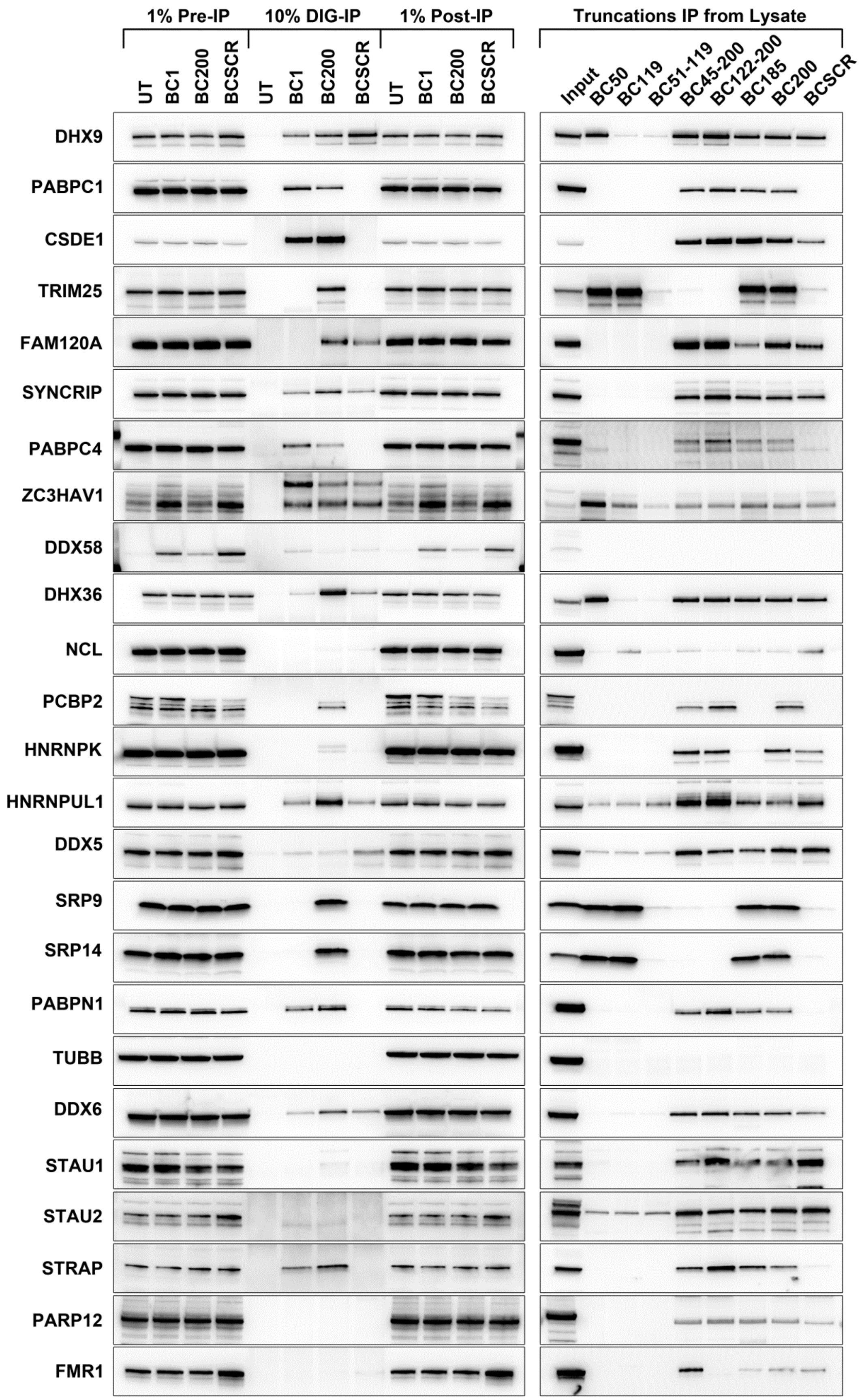

Supplementary Figure 2. Western blot analysis of 24 selected BC200 targets. Panel 1: Western blots were performed with antibodies to the indicated proteins on pull-down samples of untransfected (UT, beads alone) and transfected BC1, BC200 and BCSCR RNAs. **Panel 2:** Western blots were performed as in Panel 1 on pull-down samples of the indicated DIG-labelled RNAs incubated in 500 µL cell lysate (5 mg/mL) at a concentration of 250 nM.

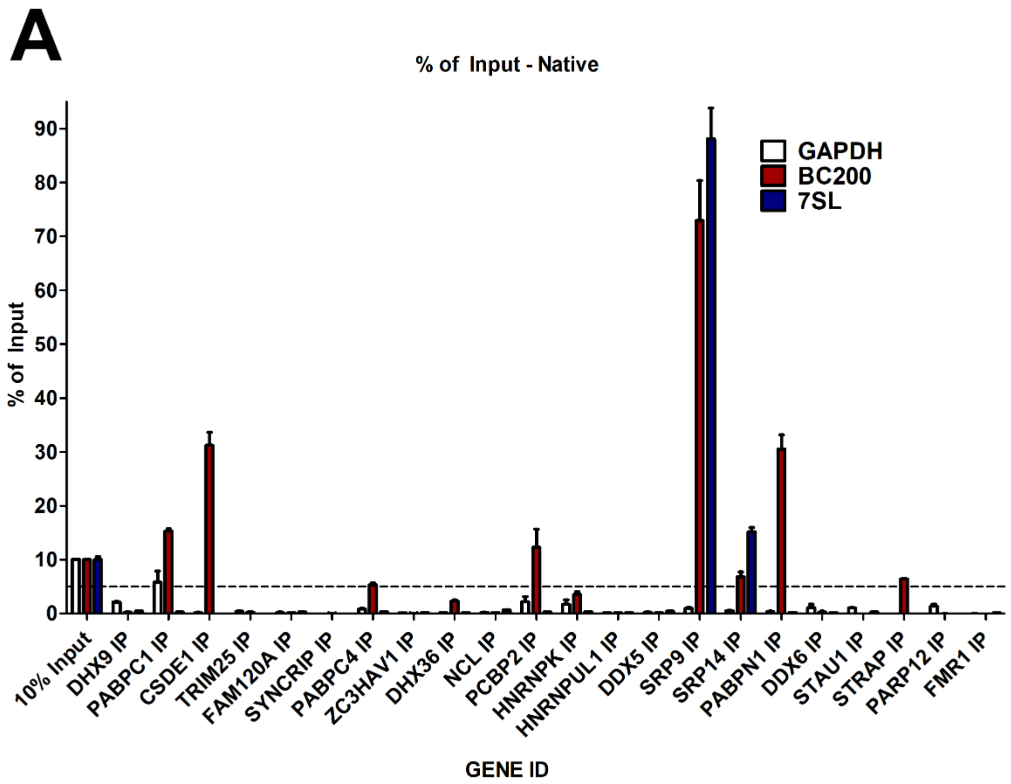

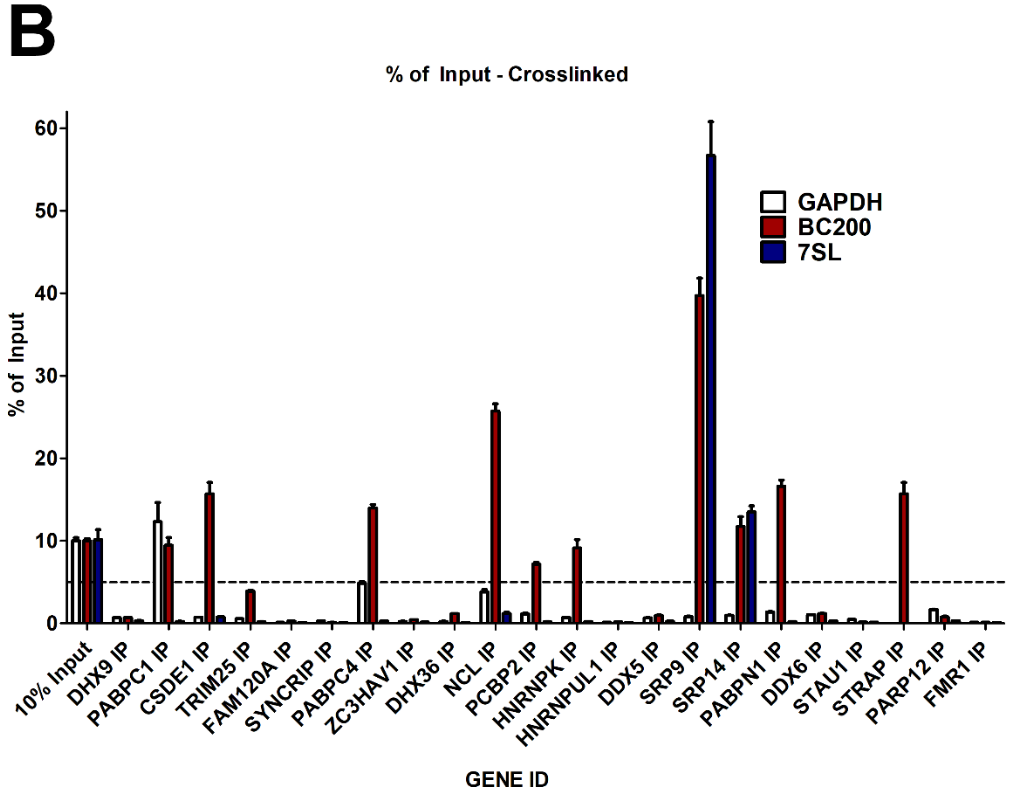

Supplementary Figure 3. RNA Immunoprecipitation experiments of 22 selected BC200 targets. (A) RT-qPCR analysis of BC200, GAPDH and 7SL enrichment by immunoprecipitation of the indicated proteins. RNA extracted from 10% of the input sample was used as a reference to calculate percent of input for each RNA that was bound to the immunoprecipitated protein. Data represents the mean of three independent replicates +/- standard deviation. Dashed line represents the threshold value of 5% input. **(B)** As in (A) however RNA immunoprecipitations were performed under conditions of formaldehyde crosslinking. Dashed line represents the threshold value of 5% input.

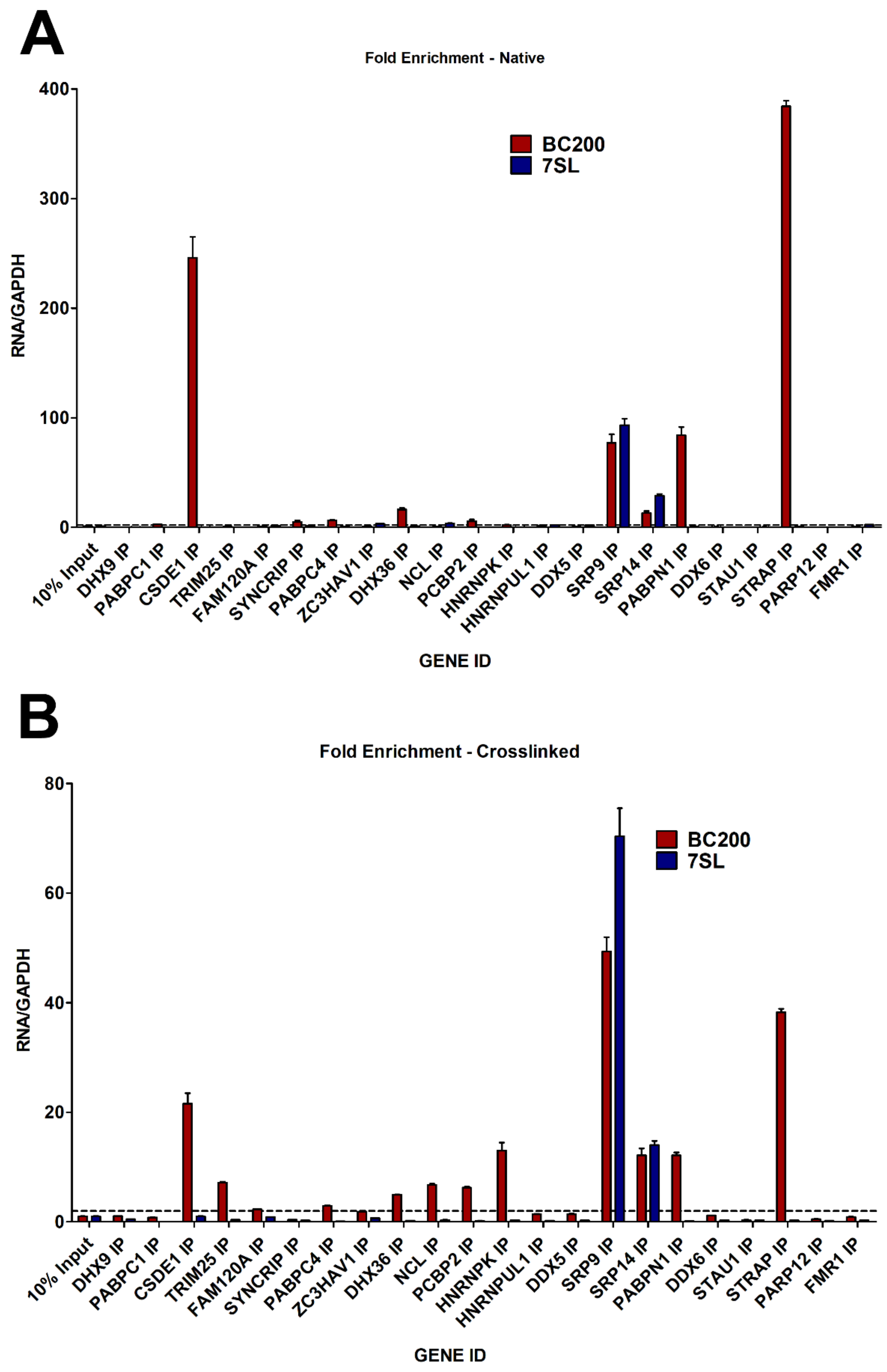

Supplementary Figure 4. RNA Immunoprecipitation experiments of 22 selected BC200 targets. (A) % Input data from Supplementary Figure 3 of BC200 and 7SL is represented relative to GAPDH to highlight specificity of the indicated interactions. Data represents the mean of three independent replicates +/- standard deviation. Dashed line represents the threshold value of 2-fold enrichment. **(B)** As in (A) however RNA immunoprecipitations were performed under conditions of formaldehyde crosslinking. Dashed line represents the threshold value of 2-fold enrichment.

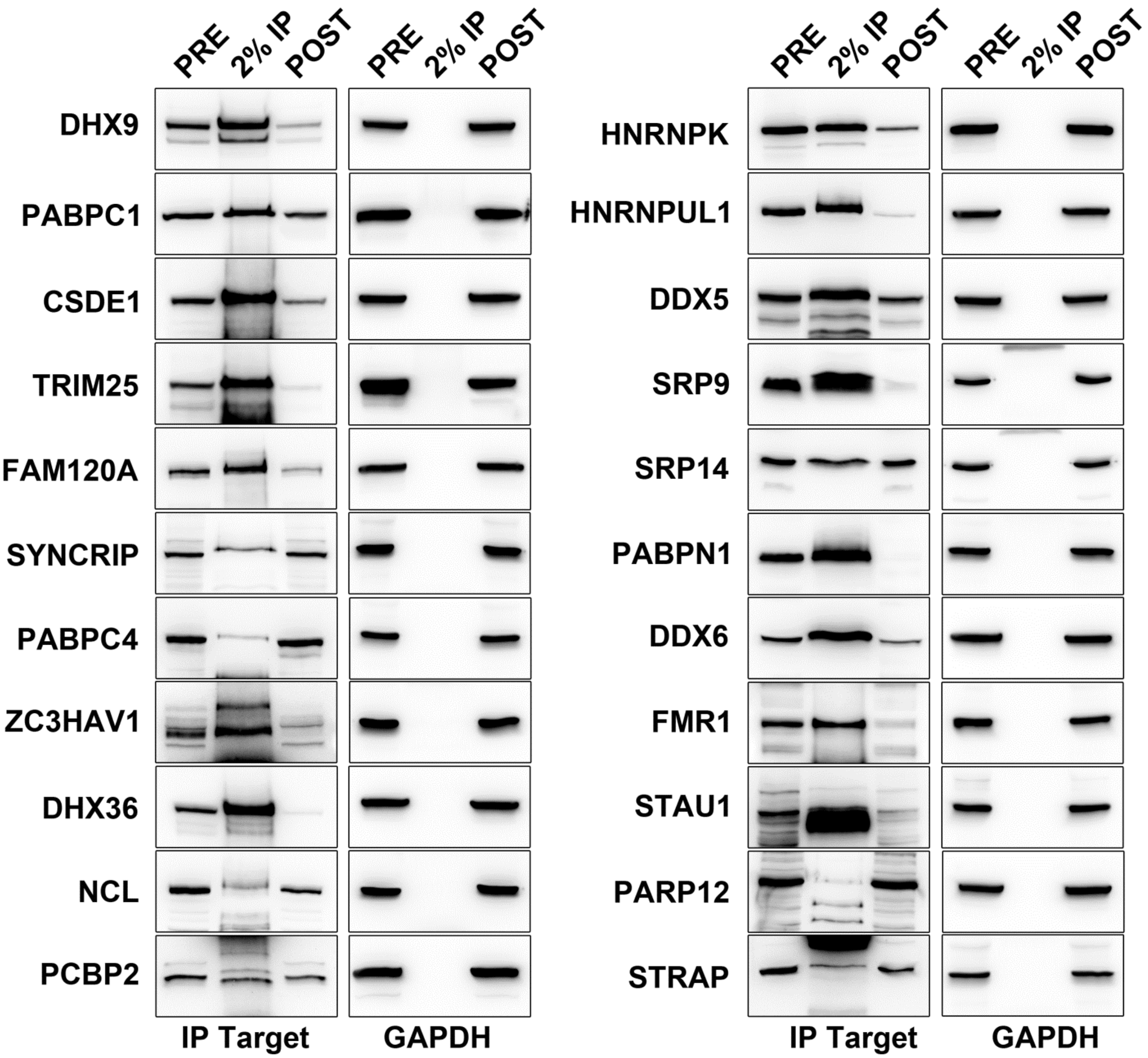

Supplementary Figure 5. Immunoprecipitation efficiency of samples from Supplementary Figure 3 and 4 was monitored by performing western blot on 50 µg of PRE and POST IP samples as well as 2% of the IP.

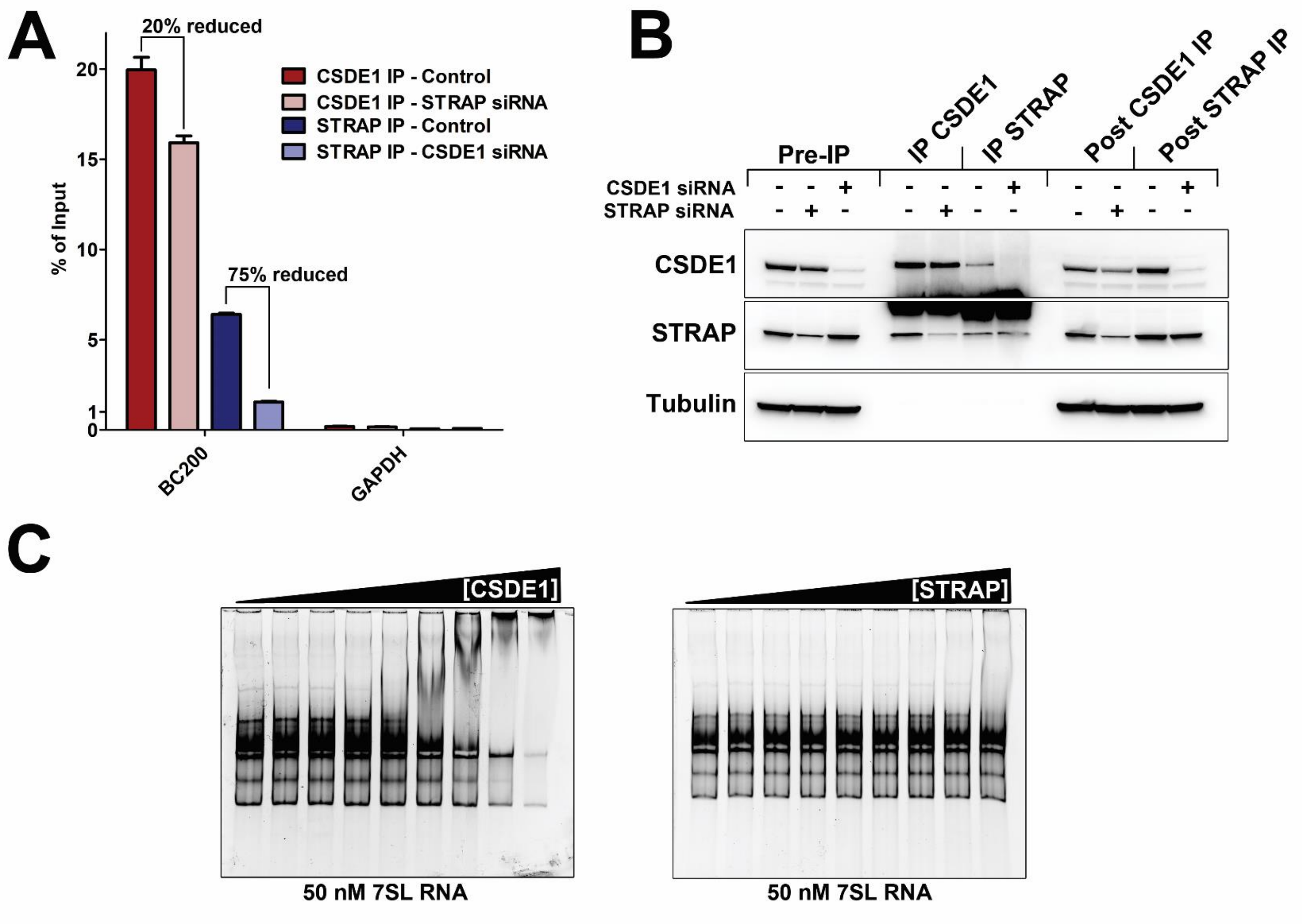

Supplementary Figure 6. CSDE1 binds directly to BC200 whereas STRAP interactions are CSDE1 dependent. (A) Immunoprecipitation experiments performed as described for Figure 5A under conditions of CSDE1 and STRAP knock-down using a second set of siRNAs (CSDE1 siRNA_1, STRAP siRNA_1, 48 hours post transfection). Co-immunoprecipitating BC200 and GAPDH RNAs were detected by RT-qPCR and compared to total RNA extracted from 10% of input. Data represents the mean of three independent replicates +/- standard deviation. **(B)** Immunoprecipitation efficiency was monitored as in Figure 4C. **(C)** Electrophoretic mobility shift assays of binding reactions prepared with 50 nM 7SL RNA and a concentration gradient of the indicated proteins. Serial dilutions of protein were used from 1000-7.8 nM. Gels were stained with SYBR Gold nucleic acid stain.

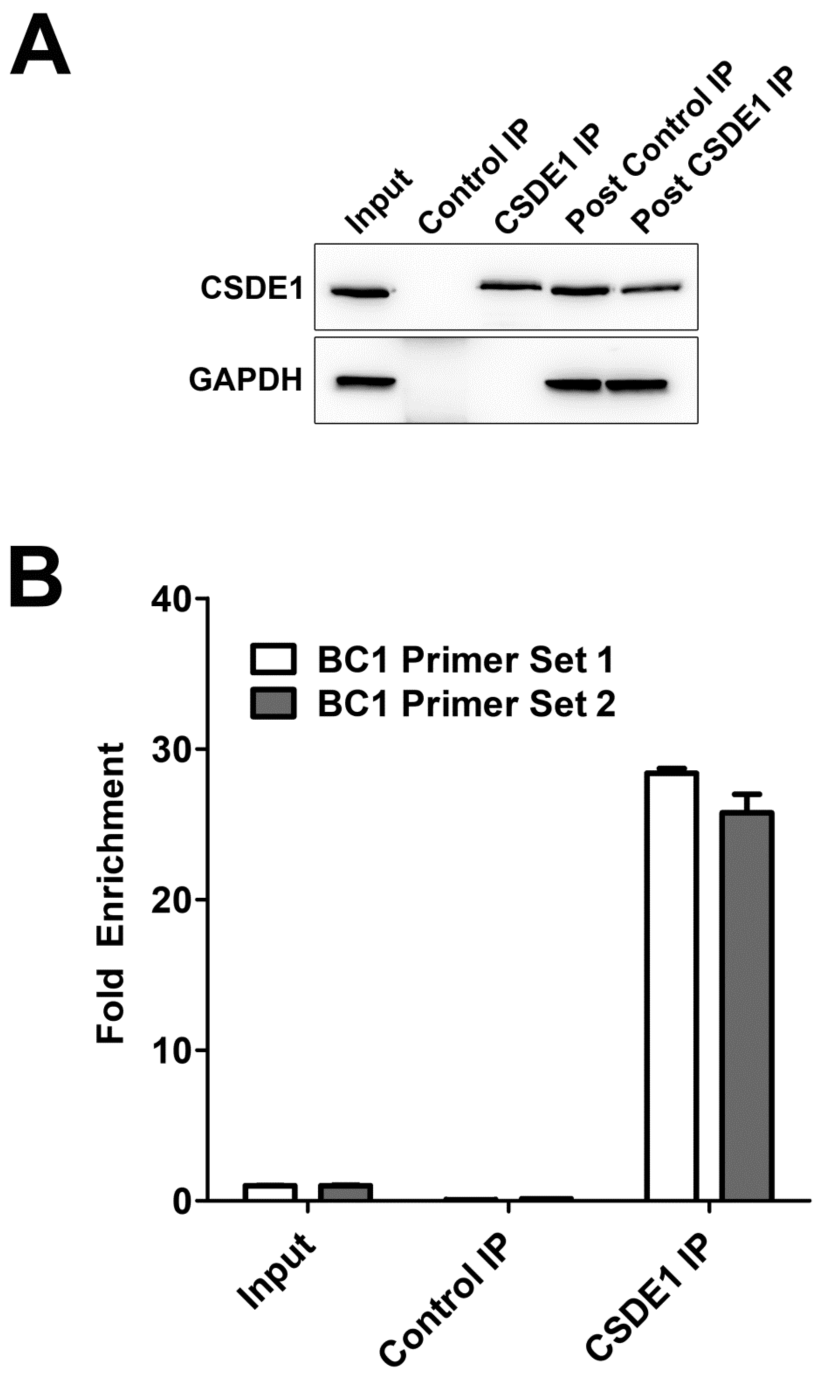

Supplementary Figure 7. (A) CSDE1 immunoprecipitation efficiency in MEF cells was monitored by performing western blot on 50 µg of PRE and POST IP samples as well as 2% of the IP. A non-targeting control antibody (Mouse isotype control ab18413, Abcam) was used to monitor specificity. **(B)** 25 ng immunoprecipitated RNA and an equal elution volume from the negative control beads were used as a templated for RT-qPCR with two primer sets specific for murine BC1. Fold enrichment was calculated relative to an equal amount of total input RNA. Data represents the mean of three independent replicates +/- standard deviation.

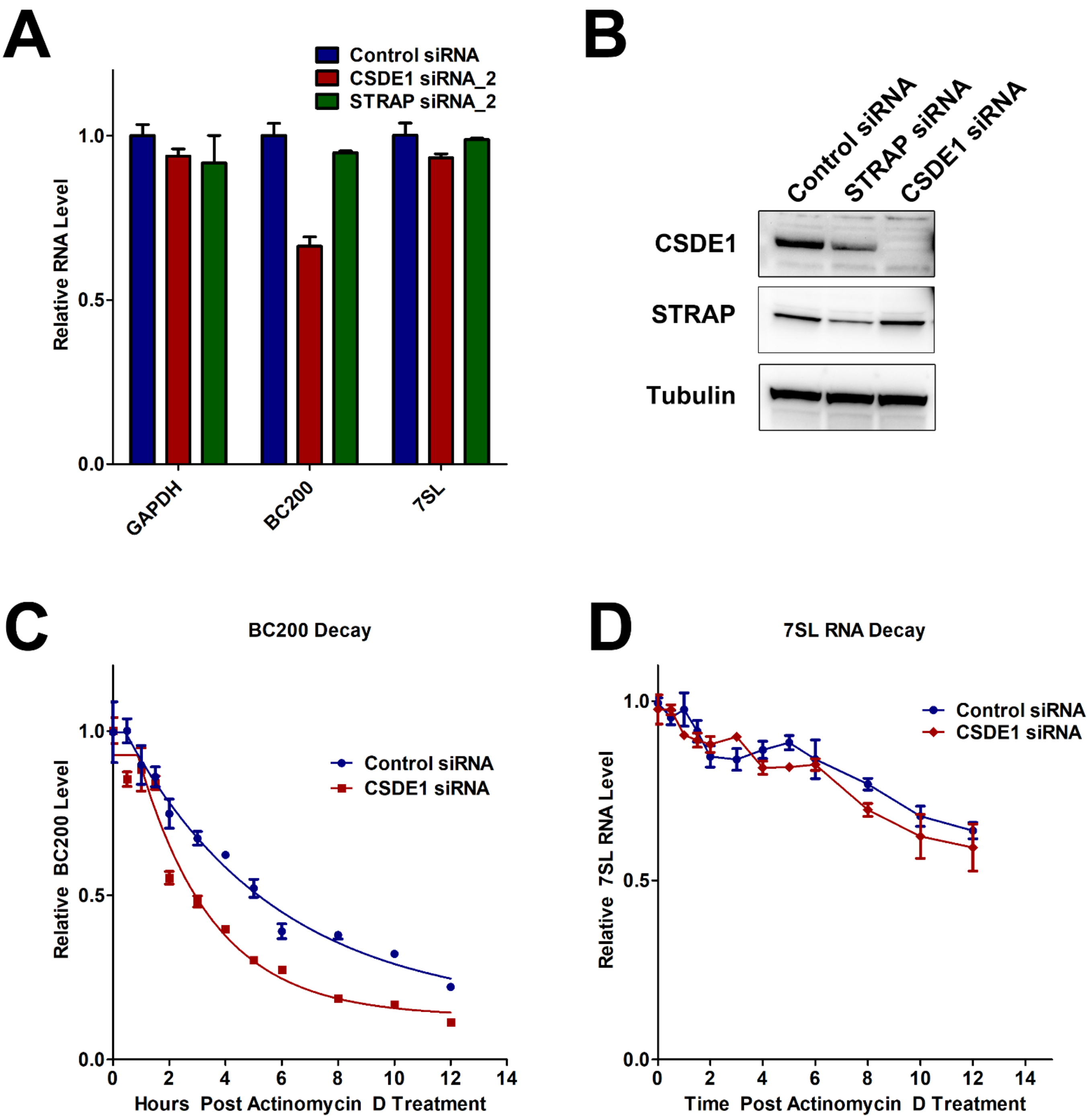

Supplementary Figure 8. (A) MCF-7 cells were transfected with a second set of CSDE1 and STRAP targeting siRNAs to confirm the data reported in Figure 6. Expression of GAPDH, BC200 and the 7SL RNA were monitored by RT-qPCR analysis following 48 hours transfection of the indicated siRNAs. **(B)** Western blot analysis of the protein levels of CSDE1 and STRAP following siRNA transfection performed in (A). **(C)** Repeat of experiment presented in Figure 7 with CSDE1 siRNA_2. BC200 decay was monitored by RT-qPCR following 72 hours CSDE1 knock-down. **(D)** As in (C), 7SL RNA decay was monitored by RT-qPCR following 72 hours knockdown of CSDE1.

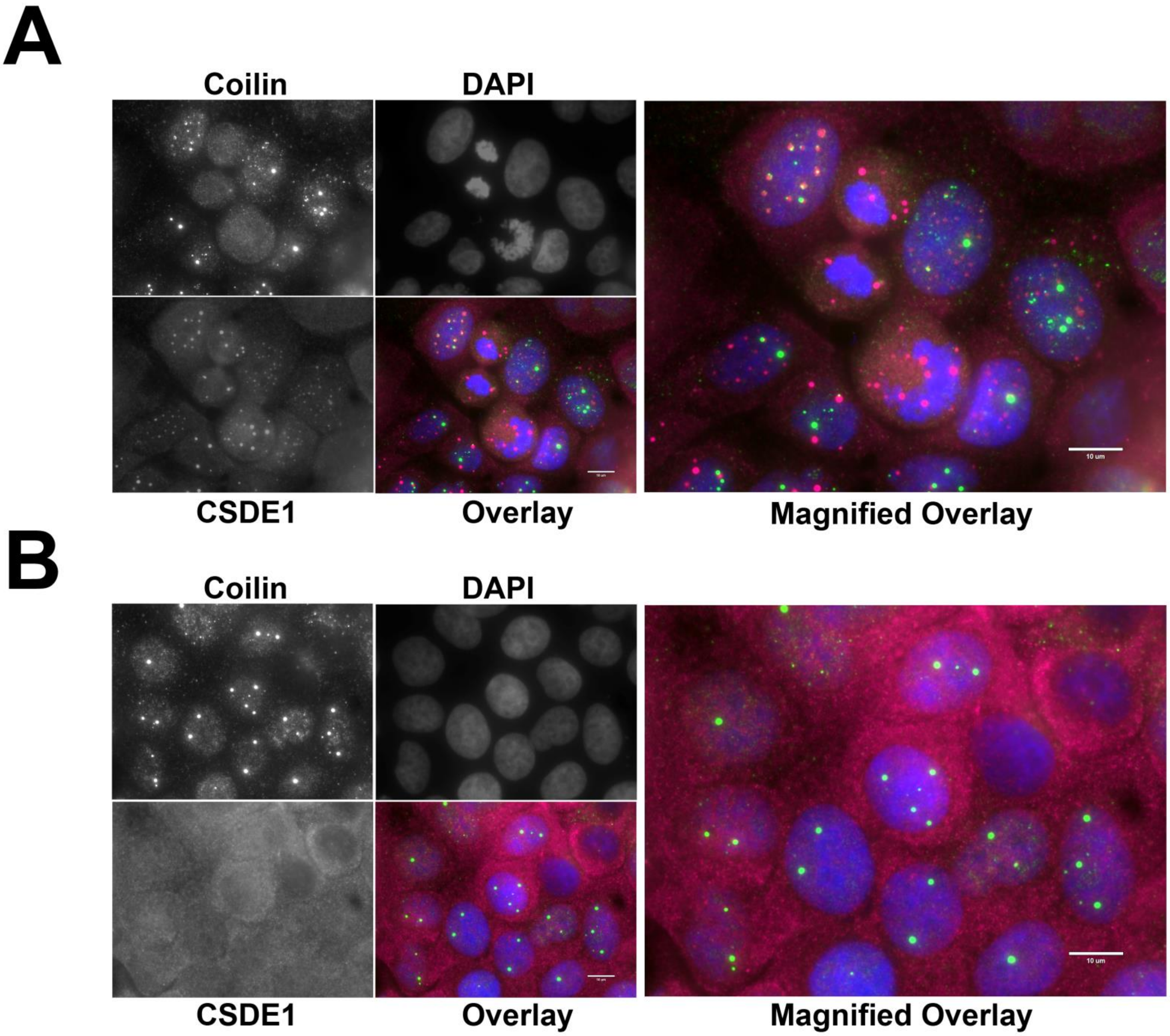

Supplementary Figure 9. CSDE1-rich foci are maintained throughout mitosis and not present in cells transfected with a control GapmeR. (A) Immunofluorescent analysis of MCF-7 cells transfected with BC200 targeting GapmeR. Cells were probed with antibodies to CSDE1 (magenta) and Coilin (green) and counter-stained with DAPI. Cells in late stage mitosis (condensed chromatin as observed with DAPI nuclear stain) are devoid of coiled bodies whereas CSDE1-rich foci remain. **(B)** Immunofluorescent analysis of MCF-7 cells transfected with non-targeting control GapmeR. Cells were probed with antibodies to CSDE1 (magenta) and Coilin (green) and counter-stained with DAPI.
